# A High-Quality Blue Whale Genome, Segmental Duplications, and Historical Demography

**DOI:** 10.1093/molbev/msae036

**Published:** 2024-02-20

**Authors:** Yury V Bukhman, Phillip A Morin, Susanne Meyer, Li-Fang Chu, Jeff K Jacobsen, Jessica Antosiewicz-Bourget, Daniel Mamott, Maylie Gonzales, Cara Argus, Jennifer Bolin, Mark E Berres, Olivier Fedrigo, John Steill, Scott A Swanson, Peng Jiang, Arang Rhie, Giulio Formenti, Adam M Phillippy, Robert S Harris, Jonathan M D Wood, Kerstin Howe, Bogdan M Kirilenko, Chetan Munegowda, Michael Hiller, Aashish Jain, Daisuke Kihara, J Spencer Johnston, Alexander Ionkov, Kalpana Raja, Huishi Toh, Aimee Lang, Magnus Wolf, Erich D Jarvis, James A Thomson, Mark J P Chaisson, Ron Stewart

**Affiliations:** Regenerative Biology, Morgridge Institute for Research, Madison, WI 53715, USA; Southwest Fisheries Science Center, National Oceanic and Atmospheric Administration (NOAA), La Jolla, CA 92037, USA; Neuroscience Research Institute, University of California, Santa Barbara, CA, USA; Regenerative Biology, Morgridge Institute for Research, Madison, WI 53715, USA; Department of Comparative Biology and Experimental Medicine, University of Calgary, Calgary, Canada; V.E. Enterprises, Arcata, CA, USA; Regenerative Biology, Morgridge Institute for Research, Madison, WI 53715, USA; Regenerative Biology, Morgridge Institute for Research, Madison, WI 53715, USA; Neuroscience Research Institute, University of California, Santa Barbara, CA, USA; Regenerative Biology, Morgridge Institute for Research, Madison, WI 53715, USA; Regenerative Biology, Morgridge Institute for Research, Madison, WI 53715, USA; University of Wisconsin Biotechnology Center, Bioinformatics Resource Center, University of Wisconsin - Madison, Madison, WI 53706, USA; Vertebrate Genome Lab, The Rockefeller University, New York, NY 10065, USA; Regenerative Biology, Morgridge Institute for Research, Madison, WI 53715, USA; Regenerative Biology, Morgridge Institute for Research, Madison, WI 53715, USA; Center for Gene Regulation in Health and Disease (GRHD), Cleveland State University, Cleveland, OH, USA; Department of Biological, Geological and Environmental Sciences, Cleveland State University, Cleveland, OH, USA; Center for RNA Science and Therapeutics, School of Medicine, Case Western Reserve University, Cleveland, OH, USA; Genome Informatics Section, National Human Genome Research Institute, Bethesda, MD 20892, USA; Laboratory of Neurogenetics of Language, The Rockefeller University/HHMI, New York, NY 10065, USA; Genome Informatics Section, National Human Genome Research Institute, Bethesda, MD 20892, USA; Department of Biology, Pennsylvania State University, University Park, PA 16802, USA; Tree of Life, Wellcome Sanger Institute, Cambridge CB10 1SA, UK; Tree of Life, Wellcome Sanger Institute, Cambridge CB10 1SA, UK; LOEWE Centre for Translational Biodiversity Genomics, 60325 Frankfurt, Germany; Senckenberg Research Institute, 60325 Frankfurt, Germany; Institute of Cell Biology and Neuroscience, Faculty of Biosciences, Goethe University Frankfurt, 60438 Frankfurt, Germany; LOEWE Centre for Translational Biodiversity Genomics, 60325 Frankfurt, Germany; Senckenberg Research Institute, 60325 Frankfurt, Germany; Institute of Cell Biology and Neuroscience, Faculty of Biosciences, Goethe University Frankfurt, 60438 Frankfurt, Germany; LOEWE Centre for Translational Biodiversity Genomics, 60325 Frankfurt, Germany; Senckenberg Research Institute, 60325 Frankfurt, Germany; Institute of Cell Biology and Neuroscience, Faculty of Biosciences, Goethe University Frankfurt, 60438 Frankfurt, Germany; Department of Computer Science, Purdue University, West Lafayette, IN 47907, USA; Department of Computer Science, Purdue University, West Lafayette, IN 47907, USA; Department of Biological Sciences, Purdue University, West Lafayette, IN 47907, USA; Department of Entomology, Texas A&M University, College Station, TX 77843, USA; Regenerative Biology, Morgridge Institute for Research, Madison, WI 53715, USA; Regenerative Biology, Morgridge Institute for Research, Madison, WI 53715, USA; Neuroscience Research Institute, University of California, Santa Barbara, CA, USA; Southwest Fisheries Science Center, National Oceanic and Atmospheric Administration (NOAA), La Jolla, CA 92037, USA; Institute for Evolution and Biodiversity (IEB), University of Muenster, 48149, Muenster, Germany; Senckenberg Biodiversity and Climate Research Centre (BiK-F), Frankfurt am Main, Germany; Vertebrate Genome Lab, The Rockefeller University, New York, NY 10065, USA; Laboratory of Neurogenetics of Language, The Rockefeller University/HHMI, New York, NY 10065, USA; Regenerative Biology, Morgridge Institute for Research, Madison, WI 53715, USA; Department of Molecular, Cellular and Developmental Biology, University of California Santa Barbara, Santa Barbara, CA 93106, USA; Department of Cell and Regenerative Biology, University of Wisconsin School of Medicine and Public Health, Madison, WI 53726, USA; Department of Quantitative and Computational Biology, University of Southern California, Los Angeles, Los Angeles, CA 90089, USA; Regenerative Biology, Morgridge Institute for Research, Madison, WI 53715, USA

**Keywords:** cetaceans, body size, evolution, conservation, developmental biology, genetic diversity, animal genomes, segmental duplications

## Abstract

The blue whale, *Balaenoptera musculus*, is the largest animal known to have ever existed, making it an important case study in longevity and resistance to cancer. To further this and other blue whale-related research, we report a reference-quality, long-read-based genome assembly of this fascinating species. We assembled the genome from PacBio long reads and utilized Illumina/10×, optical maps, and Hi-C data for scaffolding, polishing, and manual curation. We also provided long read RNA-seq data to facilitate the annotation of the assembly by NCBI and Ensembl. Additionally, we annotated both haplotypes using TOGA and measured the genome size by flow cytometry. We then compared the blue whale genome with other cetaceans and artiodactyls, including vaquita (*Phocoena sinus*), the world's smallest cetacean, to investigate blue whale's unique biological traits. We found a dramatic amplification of several genes in the blue whale genome resulting from a recent burst in segmental duplications, though the possible connection between this amplification and giant body size requires further study. We also discovered sites in the insulin-like growth factor-1 gene correlated with body size in cetaceans. Finally, using our assembly to examine the heterozygosity and historical demography of Pacific and Atlantic blue whale populations, we found that the genomes of both populations are highly heterozygous and that their genetic isolation dates to the last interglacial period. Taken together, these results indicate how a high-quality, annotated blue whale genome will serve as an important resource for biology, evolution, and conservation research.

## Introduction

The blue whale appears to be the largest animal to have ever existed (Sears and Perrin 2008). An adult can reach up to 110 feet (∼33 m) and weigh 330,000 pounds (150 metric tons) (Sears and Perrin 2008). Genomic studies of giant animals are of interest to several subfields of biomedical science. Understanding developmental mechanisms that control body size may have applications in regenerative medicine and animal husbandry. Large mammals tend to have long lives and have developed mechanisms that make them resistant to cancer, in spite of having orders of magnitude more cells (and thus more cell divisions). This puzzling phenomenon is known as Peto's Paradox (Caulin and Maley 2011; Tollis et al. 2017). Previously sequenced genomes of large animals, including other large whales, yielded some clues with regard to their possible mechanisms of cancer resistance. These include, for example, mutations and duplications of known tumor suppressors and other potentially relevant genes involved in processes such as DNA repair and apoptosis (Keane et al. 2015; Sulak et al. 2016; Vazquez et al. 2018; Tollis et al. 2019), and control over the abundance of microsatellite repeats (Park et al. 2016).

Mammalian genomes contain relatively large regions of duplicated sequence, known as segmental duplications (SDs), many of which contain genes. Copy number differences in genes inside SD loci have been reported as associated with longevity and increased body size in cetaceans (Tollis et al. 2019; Tejada-Martinez et al. 2021) and elephants (Sulak et al. 2016; Vazquez et al. 2018). In order to identify SD events that may affect mammalian development, body size, longevity, or susceptibility to cancer, we have compared the blue whale to the vaquita (*Phocoena sinus*), the world's smallest cetacean. Vaquitas, weighing up to 54.5 kg, differ from the blue whale by nearly 3,000-fold in body size. There is also a substantial difference in life span, with vaquitas living about 20 yr and blue whales living 80 to 90 yr (Hohn et al. 1996; Sears and Perrin 2008). The vaquita genome was sequenced and assembled using long-read data, enabling comparative analysis of repetitive DNA. We also computed SDs from long-read genomes assembled for bottlenose dolphin (*Tursiops truncatus*), the only other cetacean for which such data were available at the time of writing, and cattle (*Bos taurus*), for comparison as a distantly related artiodactyl (Rosen et al. 2020; Vertebrate Genomes Project 2020b; Morin et al. 2021).

The blue whale is an endangered species and included on the IUCN Red List, on Appendix I of the Convention on International Trade in Endangered Species of Wild Fauna and Flora, and on the Convention on the Conservation of Migratory Species of Wild Animals (Cooke 2018). Genomes of endangered species facilitate studies of their population structure, diversity, and demographic history, thereby aiding conservation efforts (Ming et al. 2019; Zhou et al. 2019; Morin et al. 2021; Robinson et al. 2022).

Here we report a reference-quality genome assembly and several analyses that may shed light on important aspects of blue whale biology. Our findings include a recent burst in segmental duplication activity, which resulted in amplification of several genes. We also discovered that the insulin-like growth factor 1 (*IGF1*) gene has several sites whose alleles appear to be associated with large body size in cetaceans. Finally, we show that the genomes of both Pacific and Atlantic blue whales are highly heterozygous, suggesting large, genetically diverse populations that became genetically isolated from each other during the last interglacial period.

## Results

### A Reference-Quality Genome Assembly

We generated a reference-quality genome assembly using methods developed by the Vertebrate Genome Project (VGP). The assembly is based on PacBio long reads, with Illumina/10×, Bionano optical maps, and Dovetail Hi-C used for scaffolding and polishing (Rhie et al. 2021). Manual curation resulted in multiple corrections of the computationally generated assembly, introducing 29 breaks and 64 joins between contigs within scaffolds (supplementary fig. S1, Supplementary Material online). A total of 99.8% of the assembled sequence could be assigned to 23 chromosomal-level scaffolds, which reflects the expected karyotype (21 autosomes plus *X* and *Y*). The *Y* chromosome could only be partially assembled (supplementary figs. S2 and S3, Supplementary Material online). To further improve the assembly, we applied additional false duplication removal and base accuracy polishing steps (supplementary methods, Supplementary Material online). Our assembly quality metrics are shown in Table 1. We also assembled the mitogenome, using the mitoVGP pipeline (Formenti et al. 2021) (supplementary methods, Supplementary Material online).

**Table 1:** Assembly quality metrics

Quality category	Quality metric	Computation method	Value
Input data	Sequencing technology	…	PacBio CLR
	Coverage	…	51X
	Scaffolding and polishing	…	10X, optical maps, Hi-C
Contiguity	Contig (NG50)^a^	VGP asm_stats.sh	5,559,837
	Scaffold (NG50)^a^	VGP asm_stats.sh	107,421,550
	Num gaps per Gb	VGP asm_stats.sh	329
	CC ratio	Manual calculation	42.3
Structural accuracy	False duplications	BUSCO % Duplicated^b^	1.3
	Curation improvements	N/A	Yes
Base accuracy	Base pair QV	MERQURY^c^	39.4
	k-mer completeness, primary	MERQURY^c^	94.2
	k-mer completeness, diploid	MERQURY^c^	98.3
	% of genes with frameshift mutations	GNOMON	10%
Haplotype phasing	Phased block (N50)	N50 of alt haplotype	275,582
Functional completeness	Genes, primary haplotype	BUSCO % Complete^b^	88.1
	Genes, diploid	BUSCO % Complete^b^	89.2

^a^Assuming genome size of 2,695,100,000, per flow cytometry estimate.

^b^BUSCO4, Cetartiodactyla lineage, ODB10 database. Running BUSCO in genome mode produced different scores compared to those shown in Fig. 1, which were generated by NCBI based on RefSeq annotations.

^c^Using an independent Illumina sequencing dataset from the same blue whale, which has not been used in any of the assemblies reported in this table (Vertebrate Genomes Project 2020a).

Our assembly has been annotated by NCBI (Thibaud-Nissen et al. 2013; National Center for Biotechnology Information (US) 2020) and Ensembl (Ensembl 2020). Additionally, we annotated both primary and alternate pseudohaplotype by projecting human and mouse genes using TOGA (Hiller Lab 2020a, 2020b; Kirilenko et al. 2023). We also predicted GO terms for all protein coding genes identified by the NCBI Eukaryotic Genome Annotation Pipeline using Phylo-PFP (Jain and Kihara 2019) as described in supplementary methods, Supplementary Material online: see supplementary file blue_whale.tar.gz in the Open Science Framework (OSF) repository (Jain and Kihara 2022).

**Fig. 1.**
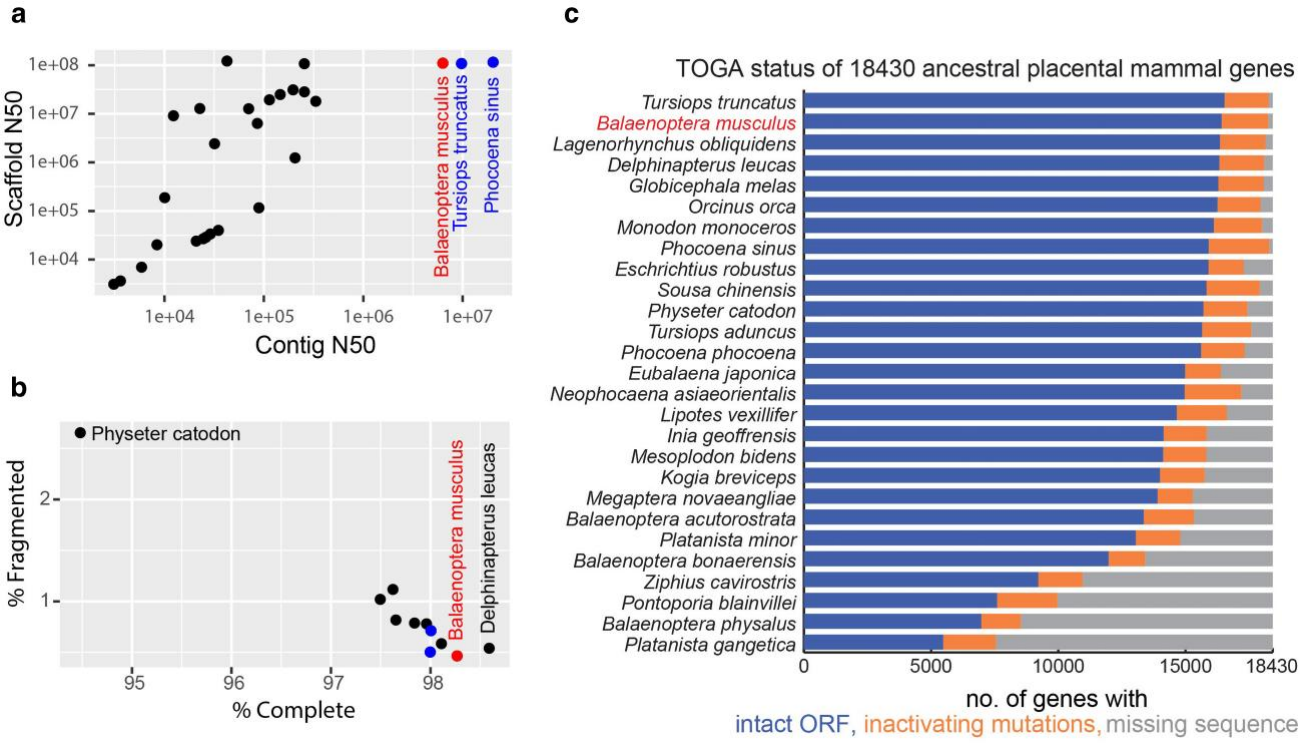
Assembly quality metrics. Blue whale (*Balaenoptera musculus*) data are shown in red; the 2 other VGP assemblies, vaquita (*Phocoena sinus*) and bottlenose dolphin (*Tursiops truncatus*), are in blue. a) Assembly contig and scaffold *N50* metrics. Contigs are segments of contiguous, i.e. gapless sequence. Scaffolds are sets of contigs that have been ordered and oriented using long-range mapping data such as optical maps and Hi-C with gaps between contigs. *N50* is a measure of average length, e.g. 50% of all bases are contained in contigs of length *N50* or longer. b) % of complete and fragmented universal single copy BUSCO orthologs found in an annotated genome. Universal single copy orthologs are genes that are present in a single copy in all or most genomes within a phylogenetic group. A high % complete score is an indication that a genome assembly is not missing a large amount of gene-coding sequence (Simão et al. 2015; Manni et al. 2021). C) TOGA status of 18,430 ancestral placental mammal genes. Note: For 2 species, different assemblies were used in panel C compared to panel A: GCA_004363415.1 instead of GCA_002189225.1 for *Eschrichtius robustus* and GCA_008795845.1 instead of GCA_023338255.1 for *Balaenoptera physalus*.

Our blue whale genome assembly is one of the highest quality of any cetacean species. Figure 1 shows data for cetacean assemblies available in the NCBI Assembly database (National Center for Biotechnology Information (US) 2022a). The blue whale and the other 2 VGP assemblies (vaquita *Phocoena sinus* and bottlenose dolphin *Tursiops truncatus*) stand out by being at least an order of magnitude more contiguous at the contig level (Fig. 1a), among the most complete and least fragmented (Fig. 1b), and having the least missing sequence and artifact inactivating mutations (Fig. 1c).

Another blue whale genome assembly, based on 10× synthetic long-read technology has recently been published (Yuan et al. 2021). It is somewhat less contiguous than our own 10×-based assembly (supplementary table S1, Supplementary Material online). However, it seems to contain fewer false duplications, as evidenced by a lower BUSCO % duplicated score, 1.7 versus 2.3 in our 10×-based assembly.

### Genome Size and Assembly Completeness

**Fig. 2.**
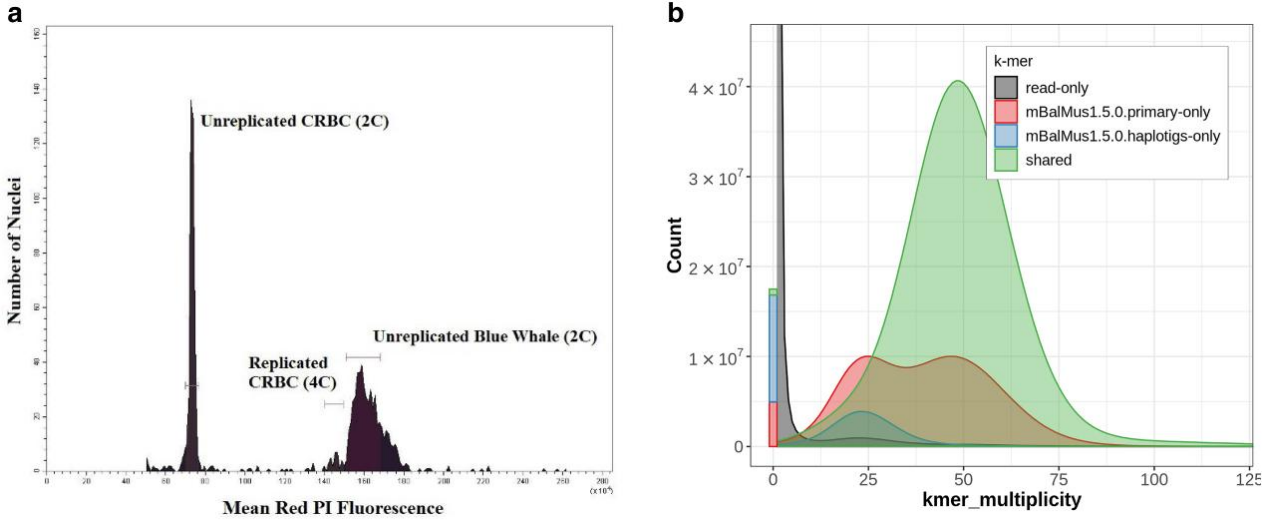
Blue whale genome size. a) Genome size estimation by flow cytometry. CRBC were used as the standard. b) K-mer spectra plot generated by the Merqury software (Rhie et al. 2020).

We estimated the genome size of the blue whale using flow cytometry, which quantifies the amount of propidium iodide (Hare and Johnston 2011) incorporated into the major groove of the nuclear DNA of a sample and a co-prepared standard. Comparing fluorescence intensity of blue whale cell nuclei to chicken red blood cell (CRBC) controls resulted in the genome size estimate of 1C = 2,695 ± 11.5 Mb (∼2.7 Gb) (Fig. 2a).

Genome assemblies tend to be somewhat smaller than genome size estimates due to the missing satellite DNA (Gordon et al. 2016). A short reads-based k-mer spectra plot produced by the Merqury software (Rhie et al. 2020) shows that only a small number of haploid k-mers are missing in the unique fraction of the genome (Fig. 2b). K-mers derived from sequencing reads of homozygous regions have an average multiplicity of around 50× coverage and match sequences of either the primary or both assembled haplotypes (“shared”). K-mers derived from reads of heterozygous regions have an average multiplicity of around 25 and match either the primary or alternate haplotype. Some of the k-mers that match neither haplotype (“read-only”) also have multiplicity of around 25, suggesting that the assembly is missing a small fraction of heterozygous k-mers, likely from the alternate haplotype. Overall, we conclude that the nonrepetitive fraction of our genome appears almost complete.

### No Evidence of Convergent Evolution for Single Nucleotide Body Size QTLs in Cetacea and Cattle

A meta-analysis of genome-wide association studies (GWAS) in cattle (*Bos taurus*), which are land relatives of cetaceans, identified 163 loci associated with body size (Bouwman et al. 2018). We used whole-genome alignments to map these loci to the blue whale and vaquita. A total of 52 loci mapped to the former and 53 to the latter, the remainder being in regions not well mapped between species. The blue whale being the largest cetacean and the vaquita the smallest, we hypothesized that blue whale genomic sequences would predominantly match alleles associated with large body size in cattle, while vaquita would match the small-body-size alleles. However this was not the case, with blue whale and vaquita matching the same bovine alleles. For example, a total of 22 loci mapped from cattle to cetaceans had nucleotides corresponding to the large-body-size allele in the latter. Sixteen of these mapped to both blue whale and vaquita and had the same nucleotide in both, while 3 mapped to each genome uniquely. Thus, both the blue whale and vaquita genome had 19 loci with a nucleotide corresponding to increased body size, which contradicts the hypothesis that loci governing size are conserved between cattle and cetaceans.

### Segmental Duplications

**Fig. 3.**
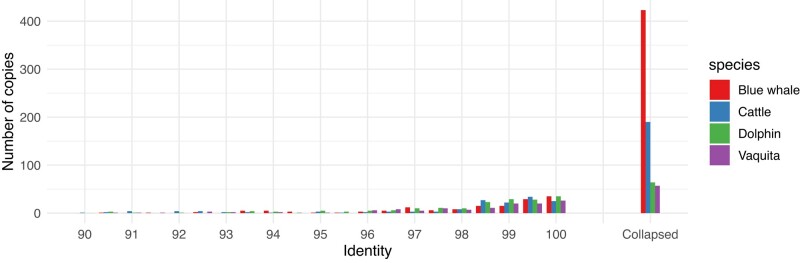
Gene duplications in blue whale, vaquita, dolphin, and cattle. Duplications that are resolved in the assembly are stratified by duplication identity. The identity of collapsed duplications may not be directly discovered from read alignments.

Previous reports of cetacean genomes have associated expansion of certain gene families through SDs with aquatic adaptation (Yim et al. 2014) and potential resistance to cancer (Tollis et al. 2019). To explore how gene expansions may relate to traits unique to the blue whale, we annotated SDs in blue whale, vaquita, and bottlenose dolphin, along with cattle as an outgroup (Fig. 3; supplementary fig. S4, Supplementary Material online). The standard definition of SD is duplicated sequences with at least 90% identity over 1 kb. Typical SD annotations exclude mobile elements and simple repeats (Bailey et al. 2001). To account for incomplete repeat masking, we also exclude sequences duplicated over 20 times in a genome. The approach to SD annotation used here was developed for vertebrate genomes and previously reported in (Toh et al. 2022). It quantifies SD using whole-genome self-alignments (Numanagic et al. 2018), and annotates duplicated gene copy numbers by excess read depth and whole-genome multimapping. We further distinguish between resolved and collapsed duplications. Resolved duplications are 2 or more distinct but highly similar segments, identified by a genome self-alignment. Collapsed duplications, a technical rather than biological term, arise when a genome assembly algorithm is unable to resolve multiple copies of a segment and the resulting assembly contains fewer copies than the genome. They are identified by excess read depth, as reads originating from multiple copies of a segment in the genome map to a single “collapsed” interval in the assembly.

Contrary to patterns of recent bursts of SD in primate (Bailey and Eichler 2006) as well as muroid genomes (Cheung et al. 2003; Thybert et al. 2018; Toh et al. 2022), SDs represent a smaller fraction of genomes in cetartiodactyla. Using the estimated mutation rate of 0.045% bp^−1^ My^−1^ (Jackson et al. 2009), both the blue whale and vaquita had relatively quiescent genomes until ∼20 M years ago. We detected a total of 20.2 Mb resolved duplications in blue whale, 30.2 Mb in vaquita, 43.6 Mb in bottlenose dolphin, and 73.2 Mb in cattle. We also identified 50.1 to 94.2 Mb of collapsed duplications in the blue whale, 15.5 to 20.8 Mb in vaquita, 29.4 to 35.0 Mb in the dolphin, and 17.6 to 22.7 Mb in cattle. Therefore, in spite of having fewer duplications resolved in the genome assembly, the blue whale shows an overall increase in duplicated bases compared to the other 3 genomes. The relatively few bases of resolved SD sequence in the blue whale assembly may reflect the sequencing depth used: 50× PacBio read coverage versus 121× in vaquita, 70× in dolphin, and ∼106× in cattle. Overall, between 2.1% to 4.8% of cetartiodactyl genomes are duplicated regions, compared to 4% to 14% in rodent and primate genomes.

SDs in the blue whale are gene rich, amounting to a roughly 7.1× burst in gene duplications relative to vaquita and dolphin, and 3.0× relative to cattle. This difference is largely driven by dramatic expansions of a limited number of genes in the blue whale. For example, the 10 most highly amplified genes account for 331 gene copies out of 700 (47%) total duplicated genes. The majority of copies of these genes could not be resolved by our genome assembly and are therefore contained in collapsed duplications (Fig. 3). The blue whale has 46 genes that have ≥4 copies, at least some of which are collapsed, compared to 8 in cattle, 9 in dolphin, and 6 in vaquita, after filtering for genes with single exons, genes annotated in pericentric DNA, and genes overlapping nuclear DNA of mitochondrial origin (supplementary table S2, Supplementary Material online).

Using algorithms developed for assembling missing segments in collapsed duplications (Vollger et al. 2019), we were able to resolve additional copies of several genes, including *KCNMB1*, *MYH8*, *DPEP2*, and *NXF2*. The assembly sizes ranged from 10 to 30 Kb, largely reflecting the longest sequence overlapping the resolved copy. The pairwise similarity of the resolved sequences ranged from 86.2% to 99.4%, indicating high sequence diversity in the missing copies of each gene (supplementary table S3, Supplementary Material online).

**Fig. 4.**
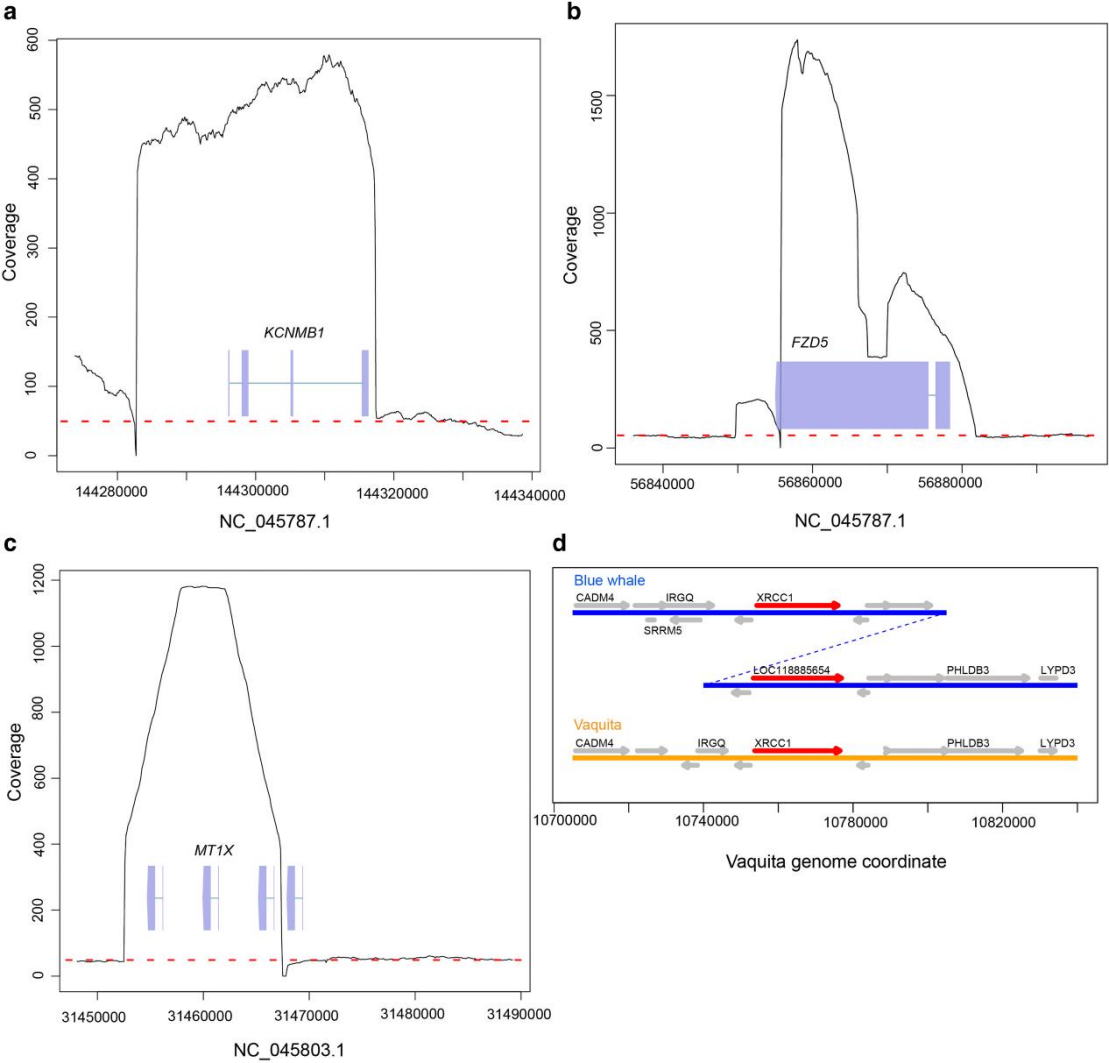
Examples of duplicated genes. a to c) Sequencing read coverage plots of the collapsed duplications containing KCNMB1, FZD5, and MT1X genes. Average coverage is shown in panels (a) to (c) in the dashed red line. MT1X duplication is partially resolved, as evidenced by the four resolved copies of the gene, shown as boxes. d) Genomic region containing XRCC1 in blue whale and vaquita. XRCC1 genes are highlighted in red and labeled by the gene name. The second XRCC1 locus in the blue whale is labeled by its locus number, LOC118885654. This locus also has an increased read coverage, suggesting an unresolved third copy; see supplementary fig. S5, Supplementary Material online.

In total, we detected 580 duplicated genes: 234 in the blue whale, 167 in the vaquita, 211 in dolphin, and 205 in cattle (supplementary table S4, Supplementary Material online). Some examples of genes duplicated in the blue whale genome are listed in Table 2 and shown in Fig. 4 and supplementary figs. S5 and S6, Supplementary Material online.

**Table 2:** Examples of genes duplicated in the blue whale

Gene (human ortholog)	Description (human ortholog)	Number of copies				Comments
		Blue whale	Vaquita	Bottlenose dolphin	Cattle	
C2orf78	Chromosome 2 Open Reading Frame 78	150	3	4	1	Uncharacterized protein. Allelic and copy number variants and a genomic rearrangement linked to cancers (Park et al. 2012; Jaratlerdsiri et al. 2017; Xicola et al. 2018). Apparent copy number in the blue whale may be affected by proximity to a centromere.
*MT1A/MT1X*	Metallothionein	53	7	1	1	Sequesters toxic metals, linked to longevity and cancer (Goulding et al. 1995; Pedersen et al. 2009; Sigel et al. 2015; Pabis et al. 2021).
*MTRNR2L5*	MT-RNR2 like 5 (pseudogene)	41	5	12	14	Humanin-like: a member of a large family of micropeptides encoded by nuclear paralogs of the mitochondrial MT-RNR2 gene.
*DPEP2*	Dipeptidase 2	32	3	3	1	Linked to body size (Deciphering Developmental Disorders Study 2015), obesity and diabetes (Liu et al. 2013; Heemskerk et al. 2015; Sárvári et al. 2021), Alzheimer's disease (Carlyle et al. 2022), cancer, immunity, and inflammation (Yang et al. 2019; Rao et al. 2021; Huang et al. 2022; Han et al. 2023)
*FZD5*	Frizzled 5	17	1	1	1	A member of the WNT signaling pathway, involved in development, regeneration, and cancer (Bhanot et al. 1996; Yang-Snyder et al. 1996; He et al. 1997; Shulman et al. 1998; Ishikawa et al. 2001; Huang and Klein 2004; Burns et al. 2008; Thiele et al. 2018; Sun et al. 2020; Dong et al. 2021)
*DDX24*	DEAD-box helicase 24	14	1	1	1	An RNA helicase involved in RNA virus-host interactions and cancer (Ma et al. 2008: 24; Ma et al. 2013: 24; Shi et al. 2016: 24)
*NCAM1* (also known as CD56)	Neural cell adhesion molecule 1	15	1	1	1	Nervous and immune systems, blood cancers (Atz et al. 2007: 1; Alegretti et al. 2011: 56; Vukojevic et al. 2020; Jennings et al. 2022: 1)
*KCNMB1*	Potassium calcium-activated channel subfamily M regulatory beta subunit 1	12	1	1	1	Linked to hypertension (Sentí et al. 2005: 1; Kelley-Hedgepeth et al. 2008: 1; Grimm et al. 2009: 1)
*XRCC1*	X-ray repair cross-complementing protein 1	2	1	1	1	Plays an important role in DNA damage repair, associated with several human cancers (Tebbs et al. 1999; Schneider et al. 2008; Hanssen-Bauer et al. 2012; Wang and Ai 2014; Ghosh et al. 2016; Meng et al. 2017; Wu et al. 2020; Demin et al. 2021; Gong et al. 2021; Tang and Çağlayan 2021).
*CDK20*	Cyclin-dependent kinase 20	4	1	1	1	A key regulator of the cell cycle, involved in mammalian development and cancer (Snouffer et al. 2017; Lai et al. 2020).
*CHRNB1*	Cholinergic receptor nicotinic beta 1 subunit	2	2	3	1	Linked to body size traits in sheep, BMI in humans. and congenital myasthenic syndromes that affect neuromuscular transmission (Müller et al. 2007; Kominakis et al. 2017; Zhao et al. 2021). The second copy has pseudogenized in vaquita.

This is a subset of genes listed in supplementary table S4, Supplementary Material online, chosen for large copy numbers in blue whale, copy number differences between blue whale and vaquita, and/or potential relevance to body size and longevity traits.

In order to identify duplication events potentially linked to body size and longevity, we selected 283 genes whose predicted copy numbers differ at least 2-fold between blue whale and vaquita (supplementary table S5, Supplementary Material online). To prioritize variants, we identified 8,649 candidate genes linked to body size, development, longevity, and susceptibility to cancer (supplementary table S6, Supplementary Material online) from published studies in whales (Tollis et al. 2019; Lagunas-Rangel 2021), dogs (Ostrander et al. 2017), cattle (Bouwman et al. 2018), and sheep (Kominakis et al. 2017), and automated literature mining engines (Kuusisto et al. 2020; Raja et al. 2020). Intersecting these 2 lists identified 133 genes of potential interest (supplementary table S7, Supplementary Material online). These included *KCNMB1*, previously reported by (Tollis et al. 2019), for which our workflow detected a collapsed duplication with 12 copies (Fig. 4a). Details of several other genes on this list linked to longevity (*MT1X*), body size (*CHRNB1, DPEP2*), development (*FZD5*, *CDK20*), cancer (C2orf78, *FZD5, DDX24, NCAM1, MT1X, XRCC1, CDK20*), obesity and diabetes (*DPEP2*), and the immune system (*NCAM1*) are available in Table 2 and Wiki pages on OSF (Bukhman et al. 2021b).

### *IGF1* Alleles Potentially Linked to Body Size in Cetaceans

Next, to further explore possible genetic bases for the large body size of the blue whale, we assessed variants of the *IGF1*, which had been reported to have large effects on body size in dogs (Ostrander et al. 2017). Domestic dogs present a unique system for the study of genetic determinants of mammalian body size due to the more than 40-fold variation in size between different breeds of the same species. Alleles of a single nucleotide within an intron of the *IGF1* gene were found to be associated with body size not only in domestic dogs, but in canines generally (Plassais et al. 2022). Therefore, we decided to investigate *IGF1* sequences in artiodactyls, including both cetaceans and their land relatives, in an attempt to discover variants that may be associated with body size in this mammalian order. To this end, we aligned whole-gene sequences and their flanking regions and carried out statistical analyses on the columns of the alignment matrix to identify sites with significant associations between adult body sizes and nucleotides across species.

**Fig. 5.**
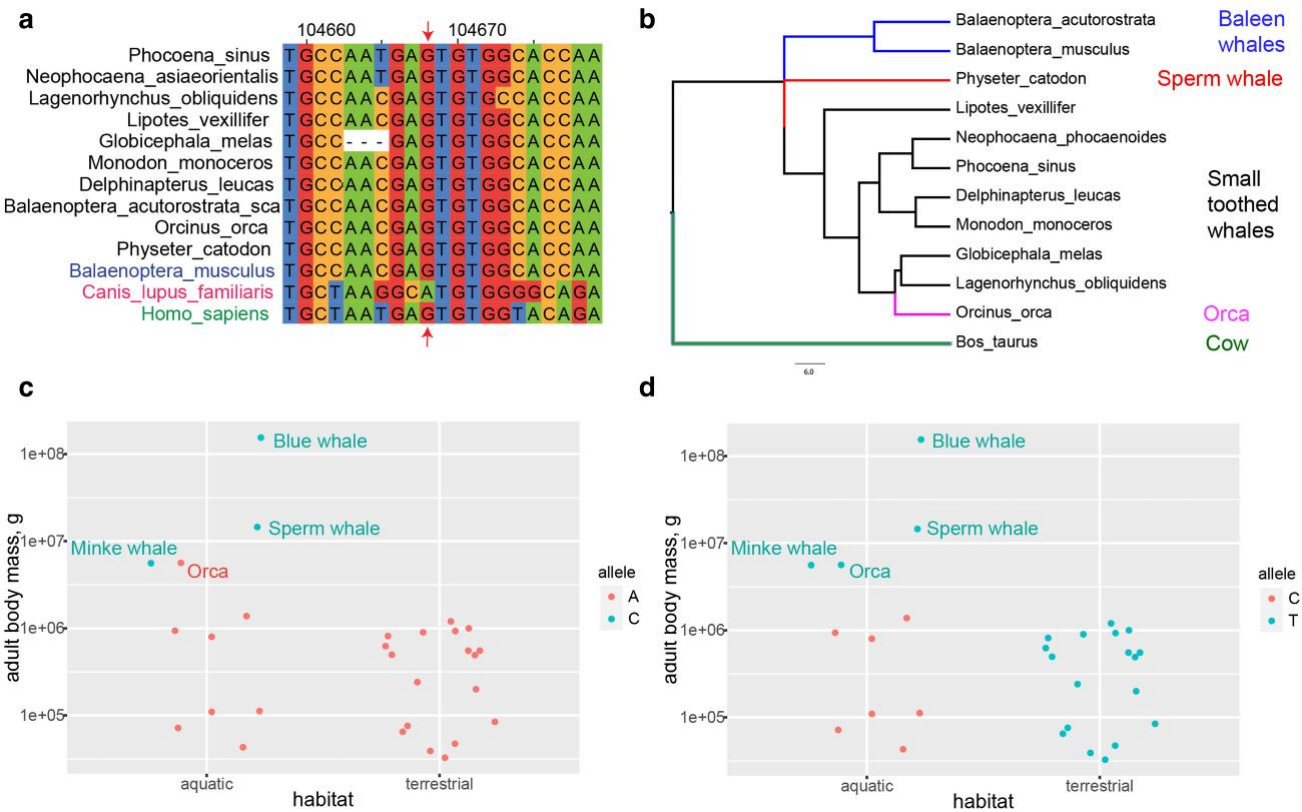
*IGF1* sites potentially associated with body size. a) Dog site rs22397284 in the context of a multiple alignment with cetaceans and the human genome. The sequences here are reverse complements of those shown in (Plassais et al. 2022), Fig. 3. rs22397284 is marked by an arrow. b) Phylogenetic tree of 11 cetaceans considered in this analysis, generated by Timetree. The 3 clades discussed in the text and the Orca are shown in different colors. c) An example type 1 site, blue whale chromosome 10 position 85,169,891. d) An example type 2 site, blue whale chromosome 10 position 85,160,822. See Table 3 for site types.

We first examined the SNP (rs22397284) in canine *IGF1* reported by (Plassais et al. 2022). This site appears to be specific to canines, with a different sequence appearing in artiodactyls, humans, and other mammals (Fig. 5a and Fig. 3 of Plassais et al. (2022)). Where canines have the CA or CG dinucleotide for large and small body size respectively, artiodactyls and other mammals have AG regardless of body size.

Our multiple alignment included whole-gene *IGF1* sequences of 11 cetacean and 18 land artiodactyl species annotated by RefSeq (Thibaud-Nissen et al. 2013). The 11 cetaceans fell into 3 phylogenetic clades (Fig. 5b): first, large baleen whales, blue and minke, *Balaenoptera musculus* and *B. acutorostrata*; second, a giant toothed whale, sperm whale *Physeter catodon*; third, smaller toothed whales, dolphins, and porpoises. The latter also includes the orca, *Orcinus orca*, the largest dolphin, as large as the minke whale.

Fitting linear models of adult body weight versus nucleotide to all columns of the *IGF1* multiple alignment matrix uncovered 2 types of potentially interesting sites. The first type is where 3 large whales, blue, minke, and sperm, have a different allele from all other artiodactyls (Fig. 5c). These sites are statistically significant when all artiodactyls are compared without regard to their habitat (linear model *P*-values of 2.5 × 10^−5^ and the false discovery rates (FDR) of 0.1 estimated using a permutation technique: see Materials and Methods). There are 2 such sites in the intergenic region upstream of the gene, 4 in the second intron, and 1 in the third or fourth intron, depending on the *IGF1* isoform (Table 3). The orca has the same allele as its smaller relatives at these sites. The second type includes sites where the four largest whales, blue, minke, sperm, and orca, have a different nucleotide compared to all smaller cetaceans. These sites appear as statistically significant when the habitat is taken into account (linear model *P-*values of 3.2 × 10^−5^ and FDR of 0.09). There are four such sites in the second intron and one in the third intron (Table 3). Interestingly, all land artiodactyls have the same variant as the large whales (Fig. 5d). Therefore, the large whales probably have the ancestral allele, while the small ones have evolved an alternative. Although the orca is more closely related to smaller dolphins, it still has the allele characteristic of baleen and sperm whales. A smaller potential ancestor of the orca is known from the fossil record (Capellini 1883; Bianucci 1996). It is possible that the orca retained the ancestral large-body allele due to incomplete lineage sorting. Additionally, the orca has a unique SINE:CHR2A_Ttr insertion in intron 2, not found in any other species in this analysis. This might be a unique adaptation compensating for the orca's lack of sites of the first type. Finally, several sites appeared statistically significant but were clearly artifacts, located in low-complexity regions where the alignment is ambiguous. These sites are not shown in Table 3. All statistically significant sites and notes are available in supplementary data, Supplementary Material online file on OSF (IGF1/hits.xlsx in (Bukhman et al. 2021b)).

**Table 3:** *IGF1* sites potentially associated with body size. Sites within the *IGF1* gene and flanking genomic regions that are significantly associated with body size in cetaceans

Site type^a^	Small cetacean allele	Large cetacean allele	Blue whale chromosome 10 coordinate	Genomic feature(s)	Multiple alignment column
1	C	T	85,170,164	Intergenic region 5′ of *IGF1*	24,474
1	A	C	85,169,891	Intergenic region 5′ of *IGF1*; LINE element L2c	24,791
2	C	T	85,160,822	Intron 2	35,626
2	G	A	85,160,446	Intron 2	36,017
1	G	C	85,150,447	Intron 2	48,867
1	A	G	85,140,848	Intron 2; LINE element L2b	62,044
1	C	T	85,140,276	Intron 2	62,965
2	C	T	85,132,164	Intron 2; LINE element L2d	78,042
2	C	T	85,126,807	Intron 2	87,228
1	G	A	85,118,402	Intron 2	99,043
2	A	G	85,112,779	Intron 3	106,724
1	C	T	85,108,387	Intron 3-4	113,202

^a^Site type 1 is where 3 large whales, blue, minke, and sperm, have a different allele from all other artiodactyls (Fig. 5c). Type 2 is where smaller cetaceans have a different allele compared to the 4 largest whales, blue, minke, sperm, and orca, and terrestrial artiodactyls (Fig. 5d).

### Historical Demography

Next, to gain insight into the history of blue whale populations, we used the pairwise sequential Markovian coalescent (PSMC) model (Li and Durbin 2011) for an analysis of historical demography. This analysis utilized reference-guided genome assemblies of Illumina sequencing data generated using our blue whale genome as the reference, with repeats masked after read alignment. We assembled Pacific blue whale Illumina sequencing reads generated by us and previously published Atlantic blue whale reads by (Árnason et al. 2018). The assemblies for the Pacific and Atlantic blue whales had 43.3× and 33.6× average depth of coverage, respectively. The PSMC plot of historical demography for the Atlantic sample was similar to the previously published analysis (Árnason et al. 2018), despite our use of a faster mutation rate (see supplementary fig. S8, Supplementary Material online for PSMC plots with alternate mutation rates). (Árnason et al. 2018) used the bowhead genome for reference-guided assembly of the blue whale and other rorqual genomes, with the maximum coalescent parameter (t) set to 20, whereas we used the de novo blue whale reference genome (repeat masked) and t = 15. We tested the effect of changing the maximum coalescent time to t = 20, and found that it had almost no effect on the plot (supplementary fig. S9, Supplementary Material online), but did reduce the number of recombination events to <10 in the last few atomic intervals in the PSMC, so we used t = 15 for all analyses reported here.

**Fig. 6.**
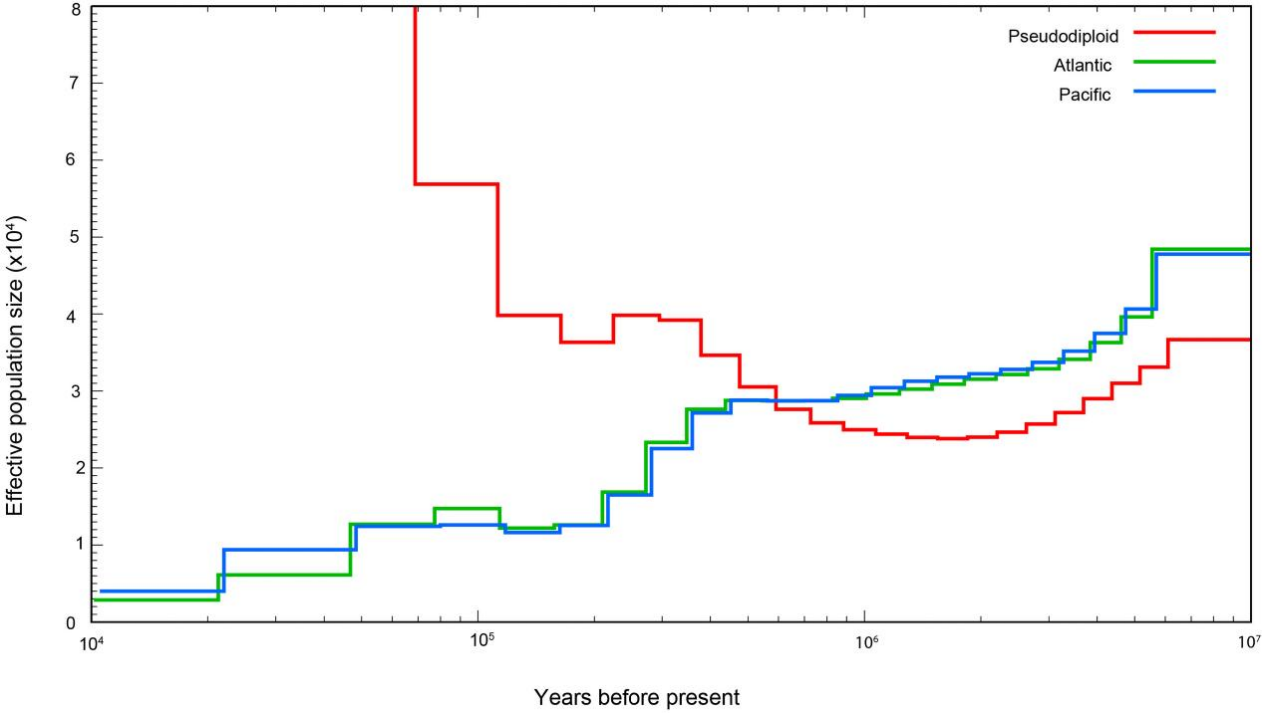
Historical demography of Pacific and Atlantic blue whales from PSMC analysis of genomes. The pseudodiploid plot represents coalescence between the 2 genomes, where the rapid increase starting approximately 125 kyr ago indicates cessation of gene flow (coalescence) between the populations. Generation time = 30.8 yr; Autosomal mutation rate (µA) = 1.58E-08 substitutions/bp/generation.

The historical demography plot of the Pacific blue whale matched that of the Atlantic blue whale until approximately 125 kyr ago, around the end of the Saalian ice age (Fig. 6). At that point, the Atlantic population appears to have grown slightly larger than the Pacific population before both ocean population sizes decreased approaching the last glacial maximum (LGM) that occurred ∼20 kyr ago; however, the apparent increase could also be due to gene flow from other blue whale populations, e.g. in the Southern Ocean. The pseudodiploid PSMC plot shows an exponential increase indicative of population division, possibly due to cessation of gene flow between the 2 ocean basins as the Eemian warm period took hold. This pattern has also been found in sperm whales from these 2 ocean basins (Morin et al. 2018).

### Heterozygosity

**Fig. 7.**
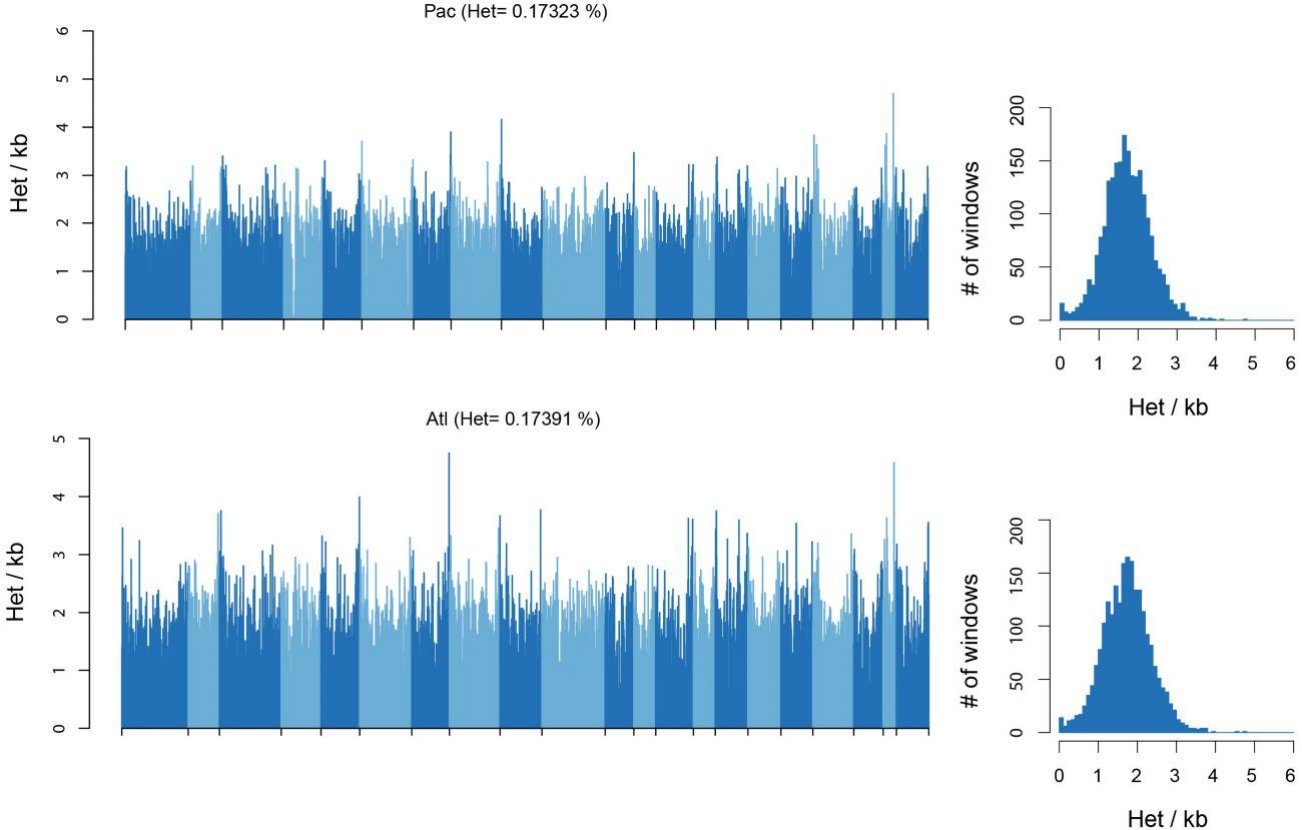
Distributions of heterozygosity across the genomes of the North Pacific and North Atlantic blue whales. (left) Barplot shows per-site heterozygosity in nonoverlapping 1-Mb windows across 22 scaffolds >10 Mb in length. Scaffolds are shown in alternating shades. (right) Histogram of the count of per-window heterozygosity levels.

The distribution of heterozygosity across the genome was determined using previously described analysis pipelines (Robinson et al. 2019). Briefly, genotypes were called and filtered from the genome assembly (above) using Genome Analysis Toolkit (GATK) (McKenna et al. 2010), filtering out loci with <1/3× or >2× mean depth of coverage, and heterozygosity was calculated as the number of heterozygous sites divided by the total number of called genotypes in nonoverlapping 1 Mb windows across each scaffold. We found high and evenly distributed levels of heterozygosity indicative of large, outbred populations (Fig. 7). See (Robinson et al. 2019) for a discussion of the effect of different demographic histories on heterozygosity.

### Runs of Homozygosity (ROH) Analysis

Runs of homozygosity (ROH) are indicative of the frequency and relative timing of inbreeding events and were frequently used to assess the impact of inbreeding on different mammalian and cetacean populations (Foote et al. 2021; Wolf et al. 2022; de Jong et al. 2023). ROH was identified by scanning the previously called genotypes using a nonoverlapping sliding window approach as implemented in DARWINDOW (de Jong 2021). We visualized heterozygosity distribution for all 23 super-scaffolds and manually altered heterozygosity thresholds, window size, and minimal window number until all visible drops in heterozygosity were marked as ROH. Using a window size of 20 kb, a minimal window number of 25, and a heterozygosity threshold of 2.5%, 108 runs of 500 kb or longer were found in both assemblies. Over 70% of these ROH were shorter than 1 Mb, indicating that most ROH were probably fragmented over time by frequent outcrossing (McQuillan et al. 2008).

**Fig. 8.**
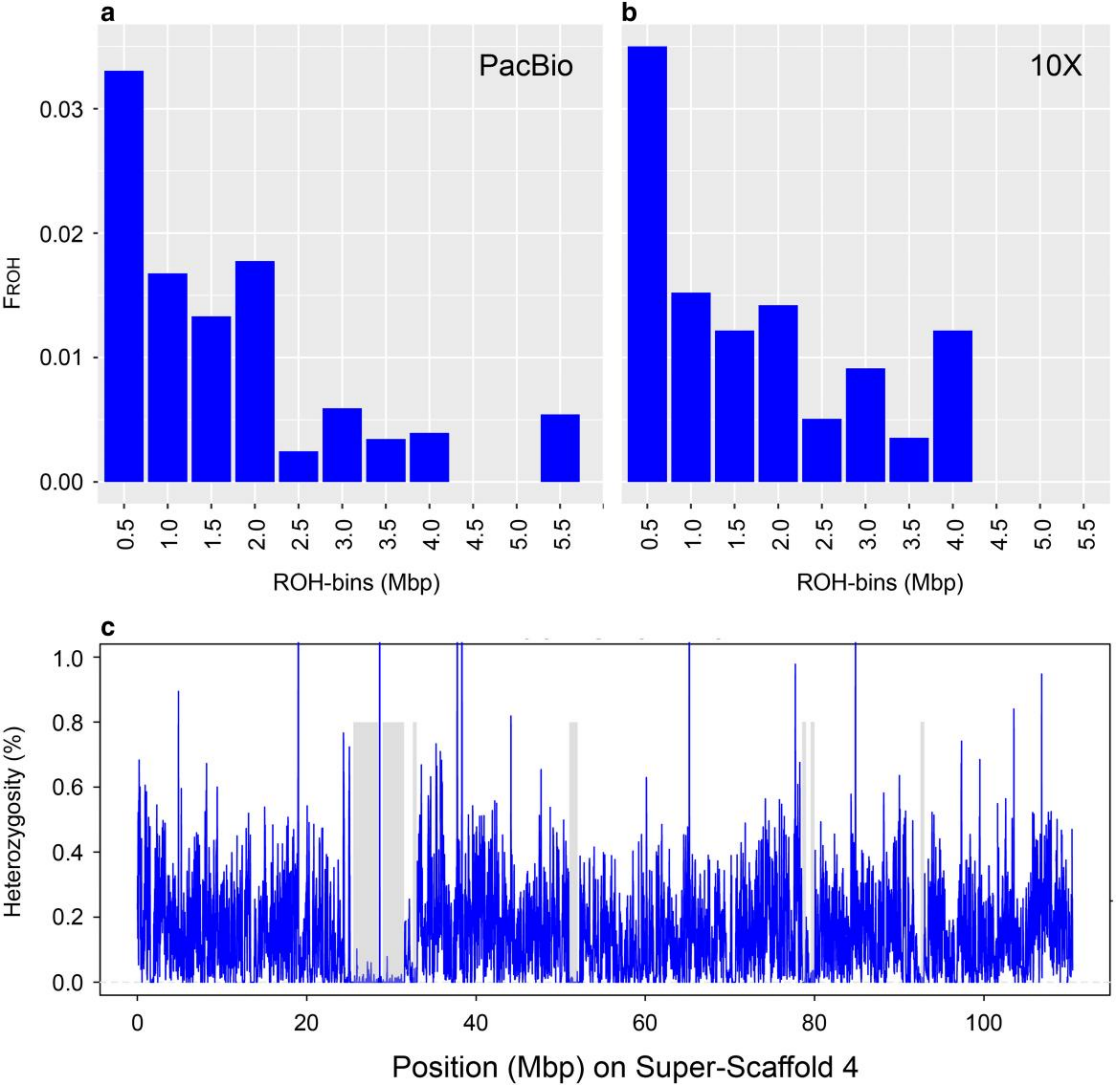
Comparison of inbreeding factors (FROH) based on the genome coverage of ROH between (a) the long-read assembly and (b) the linked-short-read assembly. ROH were identified with DARWINDOW, using a sliding-window-based approach, and sorted into respective length-bins. In both assemblies, 108 ROH over 500 kb were found; however, they appear to be more continuous in the long-read assembly as indicated by the longest ROH located on Super-Scaffold 4 (chromosome 8) of the respective assembly. A visual representation of this ROH is given in (c) and depicts the heterozygosity distribution of 20 kb windows over the scaffold in blue while identified ROH were marked as gray bars.

Compared to the long-read assembly, a slightly larger proportion of the linked-short-read assembly was covered in ROH, as indicated by a higher inbreeding coefficient, Froh (500 Kb), of 0.107 compared to 0.102 of the long-read assembly (Fig. 8a and b), respectively. The longest run of 5.5 Mb was located on super-scaffold 4 of the long-read assembly (Fig. 8c). In the linked-short-read assembly, this ROH is probably fragmented as indicated by a higher number of short (500 Kb) and medium-sized (2.5 to 4 Mb) ROHs.

## Discussion

### Genome Assembly

We have generated a reference-quality blue whale genome assembly based on PacBio long-read sequencing for contigs, and Illumina/10× linked reads, Bionano optical maps, and Dovetail Hi-C data for scaffolding and polishing. This is one of the first cetacean genomes generated in the context of the VGP, aiming at producing reference-quality genomes of all vertebrate species. We have shown that our assembly, along with 2 other VGP cetacean assemblies, are the highest quality cetacean genomes among those available from the NCBI Assembly database (National Center for Biotechnology Information (US) 2022a).

### Segmental Duplications

Previous studies of cetacean genomes have identified expansions of duplicated genes or gene families using multimapped short reads (Tollis et al. 2019) and analysis of short-read assemblies (Yim et al. 2014). High-quality genomes from long-read assemblies have been shown to resolve more duplicated sequences than short-read assemblies (Gordon et al. 2016), indicating this study enables comparative analysis of repetitive DNA and gene copy numbers in cetacean genomes. We applied a computational pipeline recently developed to analyze duplicated genes in rodentia (Toh et al. 2022) to assess if there are duplications that could be associated with size differences in cetacea. The vaquita, the world's smallest cetacean, provides a comparison point to the blue whale in terms of body size, while cattle is a terrestrial relative of both species.

A major finding of this work is the presence of large copy number expansions of a number of genes in the blue whale, while relatively few such expansions are observed in vaquita, bottlenose dolphin, and cattle. These expansions are recent in evolutionary time, making the gene copies highly similar and difficult to resolve, even when using long-read sequencing technology. Development of giant body size is also relatively recent (Slater et al. 2017; Bianucci et al. 2019), suggesting a possible link with gene expansions. However, the exact connection of these genes to body size, if any, is difficult to ascertain in the context of this study.

The majority (102/151) of duplicated gene families discovered in the blue whale were resolved by the assembly, however the majority of duplicated gene counts were identified as collapsed duplications from excess read depth (571/719), with some genes having both resolved and collapsed copies. Even with the high contiguity enabled by long-read assemblies, certain duplication architectures, in particular long tandem arrays of duplications, currently require specialized methods and data to resolve (Nurk et al. 2022).

The long-read assembly of the blue whale provides further insight into segmentally duplicated genes over a previously reported short-read analysis (Tollis et al. 2019). For example, read-depth with short reads previously identified the expansion of *KCNMB1* in blue whales (Tollis et al. 2019). Our long-read segmental duplication assembly methods were able to resolve individual copies with 0.4% to 7% divergence, indicating that the *KCNMB1* locus contains ancient (>20 Mya) duplication events. Furthermore, the long-read assembly provides additional substrate for read-depth analysis, as several additional genes of potentially high impact on body size or longevity were found to be amplified in blue whales, including vastly expanded *MT1X* and *FZD5*, additional copies of *XRCC1*, *CDK20*, and *CHRNB1*, and others.

Although this work represents a significant advance over short-read-based genome assemblies, some issues remain. We have not been able to resolve individual copies of highly amplified genes. In many cases, it was also not possible to ascertain whether duplicated gene copies form tandem arrays or are interspersed throughout the genome. Additionally, excess copies of duplicated genes were occasionally missed by our algorithm, although we were able to infer their existence from TOGA and NCBI annotations. Regarding gene copy number comparisons between species, since our workflow depends on the availability of long-read sequencing data, we have been constrained to comparing only 3 cetaceans. Such a comparison lacks statistical power and the differentially duplicated genes that we have identified should be treated as preliminary findings, rather than definitive discoveries. Finally, the biological significance of gene duplication events may vary, with greater number of copies not necessarily meaning a more important effect. Thus, in some cases, such as amylase enzymes in humans and commensal species, additional copies of a gene are fully functional and represent adaptations to factors such as diet change (Arendt et al. 2014; Pajic et al. 2019; Toh et al. 2022), in other cases, such as hemoglobin in some species of Antarctic notothenioid fishes, they are nonfunctional and ultimately lost (Bista et al. 2023). In yet other cases, such as certain TP53 and LIF retrogenes in elephants (Sulak et al. 2016; Vazquez et al. 2018), some nonfunctional duplicates are refunctionalized again, and sometimes, e.g. in case of an enzyme-turned-antifreeze protein in notothenioid fishes (Bista et al. 2023), the new function is completely different from the original. Ongoing developments in genome sequencing, assembly, and segmental duplication analysis algorithms, as well as long-read sequencing of other cetacean species, will enable better resolution of SDs and more rigorous cross-species comparisons in the near future.

### *IGF1* Sites Potentially Associated With Body Size

Alternative alleles of *IGF1* have been shown to have large effects on body size in domestic dogs (Ostrander et al. 2017). One site, located in an intron, was associated with body size in canines more generally (Plassais et al. 2022). We identified several sites within the *IGF1* gene that, while being conserved among terrestrial artiodactyls, appear to be associated with body size in cetaceans. In one set of such sites, large whales have a different allele compared to land artiodactyls and smaller cetaceans. In another, large whales have the same allele as land artiodactyls while the smaller ones have evolved an alternative. Additionally, orca, the giant dolphin, has a unique mobile element insertion not found in any other artiodactyls. It is notable that, similar to the canine site identified by (Plassais et al. 2022), all of the sites identified by our analysis are located in noncoding regions in and adjacent to the gene. The canine site is part of an lncRNA encoded on the opposite strand and possibly affecting the expression of the *IGF1* gene, rather than the sequence of the protein that it encodes. The cetacean sites identified in this study might also affect gene regulation, which could be elucidated by future research.

Although we have controlled for FDR and manually excluded sites in low-complexity regions that were obvious alignment artifacts, these results should still be treated with caution due to the large size of the whole-gene alignment, >100,000 bp, and relatively small number of species with high-quality long-read-based assemblies. Vigorous efforts are underway to generate reference-quality genomes of all cetacean species (Morin et al. 2020), which will increase statistical power for similar analyses in the near future.

### Historical Demography and Genetic Diversity

Our assembly of the North Pacific blue whale genome enabled an analysis of its population history using PSMC and a comparison to the existing Atlantic blue whale genomic data (Árnason et al. 2018) to provide insight into demography and improve granularity of blue whale taxonomy. Four subspecies of blue whales are currently recognized (Committee on Taxonomy 2020), 3 of which are found in the Southern Hemisphere and northern Indian Ocean. The fourth and nominate subspecies include blue whales in the North Atlantic and North Pacific, which have not been formally compared. Outstanding questions about subspecies designation and genetic diversity thus persist. Our PSMC analysis strongly suggests that blue whales in the North Atlantic and eastern North Pacific began to diverge 100 to 200 kyr years ago, becoming completely genetically isolated around the time of the last interglacial period. These results suggest that blue whale taxonomy should be revisited to determine whether, similar to fin and humpback whales in these ocean basins (Jackson et al. 2014; Archer et al. 2019), blue whale populations should be divided into Pacific and Atlantic subspecies in the Northern Hemisphere.

As blue whales in the North Pacific and North Atlantic diverged, the effective population sizes of both ocean basins initially remained stable before declining at ∼50 kyr ago, approaching the LGM (Fig. 6). While Antarctic blue whales use both the South Pacific and South Atlantic (Stafford et al. 2004; Branch et al. 2007a; Samaran et al. 2019; Thomisch et al. 2019), no other populations of blue whales have been confirmed to regularly use the South Atlantic. In contrast, at least 2 populations of blue whales can be found in the South Pacific year-round (Hucke-Gaete et al. 2004; Branch et al. 2007b; Galletti Vernazzani et al. 2012; Balcazar et al. 2015; Barlow et al. 2018). While less is known about the population in the western South Pacific, both morphological and genetic studies have shown similarities between the eastern South Pacific and eastern North Pacific blue whales (Gilpatrick and Perryman 2008; Durban et al. 2016; Leduc et al. 2017), suggesting they likely diverged recently. The inferred slightly larger population size followed by the sharper decline in the North Atlantic approximately 50 to 60 kyr ago (Fig. 6) may be an artifact resulting from episodic gene flow between the North Atlantic and Antarctic subspecies, ending during the approach of LGM, while the effective size in the eastern North and South Pacific declined as a metapopulation with ongoing gene flow. These demographic inferences provide insight into global gene flow and divergence patterns, with implications for taxonomy and response to climate change.

The disagreement between the pseudodiploid PSMC plot and the 2 individual PSMC plots prior to the cessation of coalescence (>100 kyr ago) also suggests that there were more complex population dynamics over time (Fig. 6), likely affected by changing population structure and levels of mixing, potentially via populations in the Southern Hemisphere. Additional genomic studies of samples from other blue whale subspecies and populations, including analysis of admixture (Foote et al. 2019) are required to further elucidate global blue whale evolutionary and demographic history.

Our analyses of heterozygosity patterns across the genome indicate that the blue whale genome is highly heterozygous and contains mostly short ROH. Even though extrapolations from a single genome to a population are challenging, we assume that this indicates a large and outbred population in the Northern Pacific, especially when compared to well-known examples of threatened or extinct populations like the mountain gorilla, the Scottish killer whale, or the Malay Peninsula rhinoceros (van der Valk et al. 2019; Foote et al. 2021; von Seth et al. 2021). Although blue whales were hunted to less than 1% of their global abundance in the late 19th to mid-20th centuries, they are believed to be recovering in all major ocean basins (Branch et al. 2007b; Pike et al. 2009; Monnahan et al. 2014). Given the separation into multiple subspecies and populations, it is probable that some populations have suffered a loss of genetic diversity and potential increase in inbreeding. While the distribution of heterozygosity across the genome of the North Pacific and North Atlantic blue whales suggests maintenance of high levels of genomic diversity, population-level genetic studies are required to assess population structure and regional inbreeding levels, especially in regions that were more heavily impacted by commercial whaling (Clapham and Baker 2018).

### Conclusion

Our high-quality chromosome-scale genome assembly of the blue whale provides more and notably more accurate genetic information for evolutionary, conservation, and other studies than previously available. Here, we used the assembly to investigate and compare SDs and other genomic features potentially important to the development of large body size, as well as to more precisely determine Pacific and Atlantic blue whale population history and genetic diversity. The assembly will serve as a valuable resource to the scientific community, enabling future comparative genomics studies to further our understanding of large animal longevity and associated resistance to cancer, and helping conserve this magnificent species.

## Materials and Methods

### Genome Size Estimation by Flow Cytometry

The genome size of the blue whale was estimated using nuclei isolated from blue whale fibroblast cell culture BW-04. The DNA amount per nucleus was estimated using flow cytometry following methods described in (Johnston et al. 2019). In brief, whole cells from the blue whale cell culture were placed into 1 mL of ice-cold Galbraith buffer in a 2 mL Kontes Dounce grinder along with a single drop of commercially prepared chicken red blood cells (CBRCs). The nuclei of sample and standard were released by grinding the mixture using 12 strokes of the A pestle at a rate of 12 strokes in approximately 9 s. Nuclei released by the grinding were passed through a 40 µm nylon mesh, stained with 25 µL/mg propidium iodide, and held in the dark and cold for 3 h. The relative red (PI) fluorescence of the 2C nuclei from the sample and standard were scored to channel number using a Beckman Coulter CytoFLEX flow cytometer. The 1C amount of DNA in the blue whale was determined as the ratio of the 2C red fluorescent mean 2C peak positions of the sample and standard times the 1C amount of DNA in the standard. Because the commercial CRBCs are supplied with no information on the sex or strain of the chicken, the genome size of the CBRCs was scored by multiple co-preparations of CBRC and lab strains standards, *Drosophila virilis* (1C = 328 Mb) and *Callosobruchus maculatus* (1C = 1,205 Mb). The resulting estimate of the CRBC genome size was 1C = 1,250 Mb, consistent with published values for a white leghorn rooster.

### Construction and Sequencing of High Molecular Weight Genomic DNA Libraries Using PacBio Technology

HMW DNA was prepared according to the Pacific Biosciences 20 kb gDNA library protocol (Pacific Biosciences). The quality and quantity of the finished libraries were assessed using an Agilent Fragment Analyzer and Qubit® dsDNA HS Assay Kit, respectively. Libraries were sequenced on one Pacific Biosciences Sequel 1 M SMRT Cell for a 10-h movie.

### VGP Genome Assembly Workflow

The blue whale VGP genome was assembled using version 1.5 of the standard VGP assembly workflow described in (Rhie et al. 2021). Briefly, PacBio genomic reads were assembled using Falcon. The assembly was then partially phased using Falcon-unzip, bases polished using Arrow, and false duplications purged using purge_haplotigs. This was followed by successive scaffolding with: 10× linked-read data using 2 rounds of scaff10x; Bionano optical maps using Solve; and Dovetail Hi-C data using Salsa2. The scaffolded assembly was polished using Arrow using the CLR long reads and 2 rounds of freebayes using the 10× linked reads. Assembly assessment, decontamination, manual curation, and assignment of chromosomal-scale scaffolds was performed as previously described (Howe et al. 2021) using gEVAL (Chow et al. 2016).

### Segmental Duplications Analysis

Segmental duplications and gene copy numbers were defined using a combination of self-alignments and read depth defined in the Segmental Duplication Annotation pipeline (Chaisson 2023). The boundaries of collapsed duplications identified from excess read depth are annotated using a hidden Markov model (HMM) trained on single-molecule sequencing read depth. The copy number of a collapsed duplication is calculated from the average depth in each collapsed region, for collapses greater than copy number 6, or from the HMM for collapses at or below this copy number. Genes in collapsed duplications were identified as genes that fully overlap intervals identified as collapse.

This pipeline identifies duplicated genes by first mapping human Gencode v29 sequences using minimap2 -x splice (version 2.17-r941), extracting the sequence of the entire gene body, and re-mapping using minimap2 allowing for multiple alignments of a gene. Genes with a single exon were excluded. In order to identify genes that conform to the definition of a segmental duplication, duplicated gene copies are identified as sequences with at least 90% identity and 90% similar gene length.

The SDA method was used to assemble missing collapsed duplications (Vollger and Chaisson 2022). A coverage of 40 was specified, and the method was used with the “–collapse” option.

### Identification of Genes Linked to Body Size and Related Traits

We identified genes linked to body size, development, longevity, and susceptibility to cancer by manual review of published studies in whales (Tollis et al. 2019; Lagunas-Rangel 2021), dogs (Ostrander et al. 2017), cattle (Bouwman et al. 2018), and sheep (Kominakis et al. 2017). We also used KinderMiner (Kuusisto et al. 2020) and SKiM (Raja et al. 2020) literature mining systems to identify genes linked to relevant search terms, including “developmental clock”, “body size”, “dwarfism”, “gigantism”, “longevity”, “cancer”, “growth”, and “overgrowth”. We also searched a curated gene-disease database for genes linked to dwarfism. See our OSF repository (Bukhman et al. 2021a) for a more detailed description in wiki/1_body_size_and_development and the R script 1_body_size_and_development/body_size_and_development_genes.R. See also (Toh et al. 2022) for a more detailed description of our literature mining methodology.

### *IGF1* Multiple Alignment and Statistical Analysis of the Alignment Matrix

We made use of NCBI e-utilities (National Center for Biotechnology Information (US) 2010; Winter 2017; Bukhman 2022a) to retrieve genomic sequences of entire *IGF1* genes plus 10 Kb of flanking sequence on both sides. The retrieved sequences are available on OSF (Bukhman 2022b). The alignment of 29 artiodactyl, dog, and human genomes was performed using clustalw2 with the following command: clustalw2 -MAXSEQLEN = 200,000 -ALIGN -TREE -QUICKTREE -TYPE = DNA -KTUPLE = 4 -TOPDIAGS = 2 -WINDOW = 4 -INFILE = IGF1_genomic_seqs.fna. The resulting alignment is available on OSF (Bukhman 2022c: 1). The rows of the alignment were manually sorted by habitat and adult body mass using Jalview (Waterhouse et al. 2009). The sorted alignment is available on OSF (Bukhman 2022d). We fitted 2 linear models for each column of the alignment matrix, modeling the logarithm of adult body size as a function of the nucleotide alone or the nucleotide and the habitat. The latter was defined as “aquatic” for cetacea or “terrestrial” for land-dwelling artiodactyls. The R markdown notebook of this analysis is available on GitHub (Bukhman 2022e). Adult body mass values were retrieved from PANTHERIA and other sources (Jones et al. 2009; Treseus 2022; Wikipedia contributors 2022a; Wikipedia contributors 2022b; Wikipedia contributors 2022c; Wikipedia contributors 2022d; Castillo-Ruiz).

FDRs were estimated by randomly permuting species labels of the body mass values. All values were permuted in the first version of the analysis, which did not account for the habitat. Values were permuted separately within terrestrial and aquatic groups for the second version of the analysis. FDRs for different *P*-value thresholds were estimated by dividing the average number of hits in permuted datasets by the number of hits in the original dataset. Each analysis included 2 runs of 1,000 random permutations each, in order to verify the stability of the FDR estimates. R scripts, *P*-values of alignment positions, and FDR estimates are available on OSF (Bukhman et al. 2021b) in folder IGF1/body-size-loci_try2_artiodactyls-etc/4_permut-fdr.

### PSMC and Genome Heterozygosity

We used PSMC (Li and Durbin 2011) for analysis of historical demography. Illumina 150 bp (Pacific) or 100 bp (Atlantic) paired-end reads were trimmed with the BBduk function of BBTools (Bushnell) for average quality (q ≥ 20), 3′ ends trimmed to q ≥ 15, and minimum length (≥40 nucleotides). Trimmed reads were aligned to a previously generated reference blue whale mitogenome, GenBank accession MF409242.1 (Árnason et al. 2018), using BWA mem (Li and Durbin 2009), and the unmapped reads exported as reads representing only the nuclear genome. Nuclear reads were aligned to the reference genome primary pseudohaplotype, NCBI Assembly accession GCF_009873245.2, also using BWA mem. Duplicate reads were removed using Picard Tools v2.20 (Broad Institute) and the resulting genome alignments from one library (Atlantic sample) and 2 combined libraries (Pacific sample) were assessed for average depth of coverage using ANGSD (Korneliussen et al. 2014). From each assembly, the diploid genome was extracted using SAMtools (Li et al. 2009) following (Morin et al. 2018), with minimum and maximum depth of coverage set to 1/3× and 2× the average depth of coverage for each assembly, and PSMC run with generation time of 30.8 yr (Taylor et al. 2007), and an autosomal mutation rate (µA) of 1.58E-08 substitutions/bp/generation, based on mean rate of 5.14E-10 substitutions/bp/year for mysticetes (Jackson et al. 2009), with 100 bootstrap resamplings on all PSMC analyses. The PSMC parameters were left as the default values used for humans (Li and Durbin 2011), and we verified that, after 20 rounds of iterations, at least ∼10 recombinations were inferred to occur in the intervals each parameter spans: *P* = (4 + 25*2 + 4 + 6) (Li and Durbin 2011). After the generation of the PSMC results, data were plotted with multiple mutation rates to test the effect of mutation rate uncertainty.

A pseudodiploid genome was made by randomly sampling an allele at each site from each of the genome assemblies from the 2 ocean basins using seqtk (Li 2022), followed by PSMC analysis using the same parameters as above, as previously described (Morin et al. 2018; Foote et al. 2019). The pseudodiploid PSMC analysis provides information on changes in the rate of coalescence between 2 individual genomes through time, and, therefore, the timing of changes in population structure relative to the changes in effective population size inferred by the single-genome PSMC analyses (Cahill et al. 2016; Chikhi et al. 2018).

The distribution of heterozygosity across the genome was determined using previously described analysis pipelines (Robinson et al. 2019). Briefly, genotypes were called and filtered from the genome assembly (above) using GATK (McKenna et al. 2010), filtering out loci with <1/3× or >2× coverage, and heterozygosity was calculated as the number of heterozygous sites divided by the total number of called genotypes in nonoverlapping 1Mb windows across each scaffold.

## Supplementary Material

Supplementary material is available at *Molecular Biology and Evolution* online.

## Supplementary Material

msae036_Supplementary_Data

## Data Availability

Raw sequencing and mapping data and various versions of genome assemblies are available from VGP GenomeArk (Vertebrate Genomes Project 2022a: 1; Vertebrate Genomes Project 2022b). Primary pseudohaplotype genome assembly and annotation are available from NCBI Genome, id 7017 (National Center for Biotechnology Information (US) 2022b). Primary pseudohaplotype assembly is mBalMus1.pri.v3, RefSeq accession GCF_009873245.2, and GenBank accession GCA_009873245.3. Alternate pseudohaplotype assembly is mBalMus1.alt.v2, GenBank accession GCA_008658375.2. Mitochondrion assembly generated in this study has GenBank accession CM018075.1. Fibroblast transcriptomics data have been deposited to the NCBI SRA archive. Iso-seq accession SRX6360705, Illumina RNA-seq accession SRX7696402. Additional supplementary materials and datasets are available on OSF (Bukhman et al. 2019). Whole genome annotations are available from NCBI RefSeq (National Center for Biotechnology Information (US) 2020), Ensembl Rapid Release (Ensembl 2020), and TOGA (both haplotypes) (Hiller Lab 2020a; Hiller Lab 2020b).

## Code Availability

All software used in this study is in the public domain, with the sole exception of Dovetail HiRise. VGP software repository is available on GitHub (Vertebrate Genomes Project 2022c). Y.V.B.'s R scripts and R Markdown notebooks are likewise available on GitHub (Bukhman 2022f). References to other software repositories are given in the Methods.
