# Supplementary material for: A High-Quality Blue Whale Genome, Segmental Duplications, and Historical Demography
Source: Mol Biol Evol. 2024 Feb 20;41(3):msae036. doi: 10.1093/molbev/msae036 (PMC10919930; doi:10.1093/molbev/msae036)
Supplement: msae036_Supplementary_Data [file msae036_supplementary_data.zip › Blue whale genome paper supplement.pdf]

Supplement: A high-quality blue whale genome, segmental duplications, and historical demography

Yury V. Bukhman, Phillip A. Morin, Susanne Meyer, Li-Fang Chu, Jeff K. Jacobsen, Jessica Antosiewicz-Bourget, Daniel Mamott, Maylie Gonzales, Cara Argus, Jennifer Bolin, Mark E. Berres, Olivier Fedrigo, John Steill, Scott A. Swanson, Peng Jiang, Arang Rhie, Giulio Formenti, Adam M. Phillippy, Robert S. Harris, Jo Wood, Kerstin Howe, Bogdan M. Kirilenko, Chetan Munegowda, Michael Hiller, Aashish Jain, Daisuke Kihara, J. Spencer Johnston, Alexander Ionkov, Kalpana Raja, Huishi Toh, Aimee Lang, Magnus Wolf, Erich D. Jarvis, James A. Thomson*, Mark J.P. Chaisson*, Ron Stewart**

*Corresponding authors

Supplementary figures

Manual curation

A.

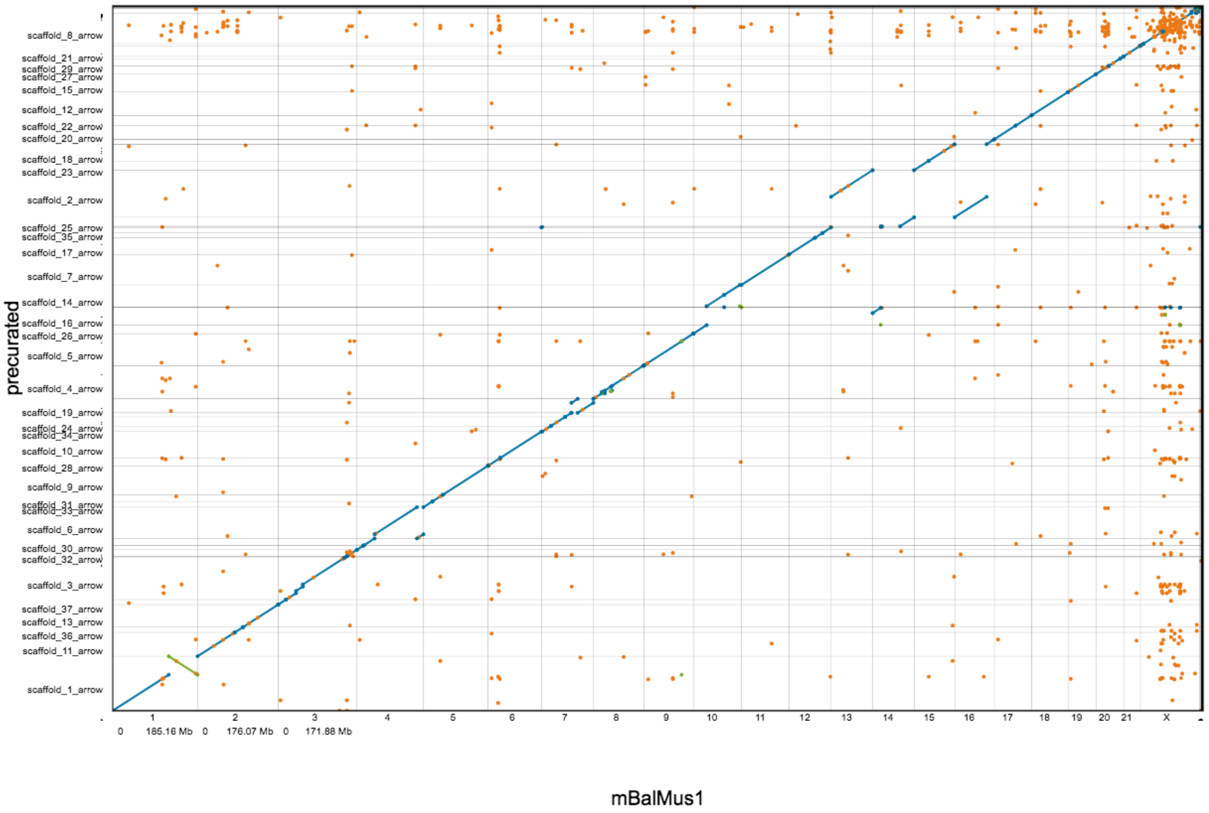

B.

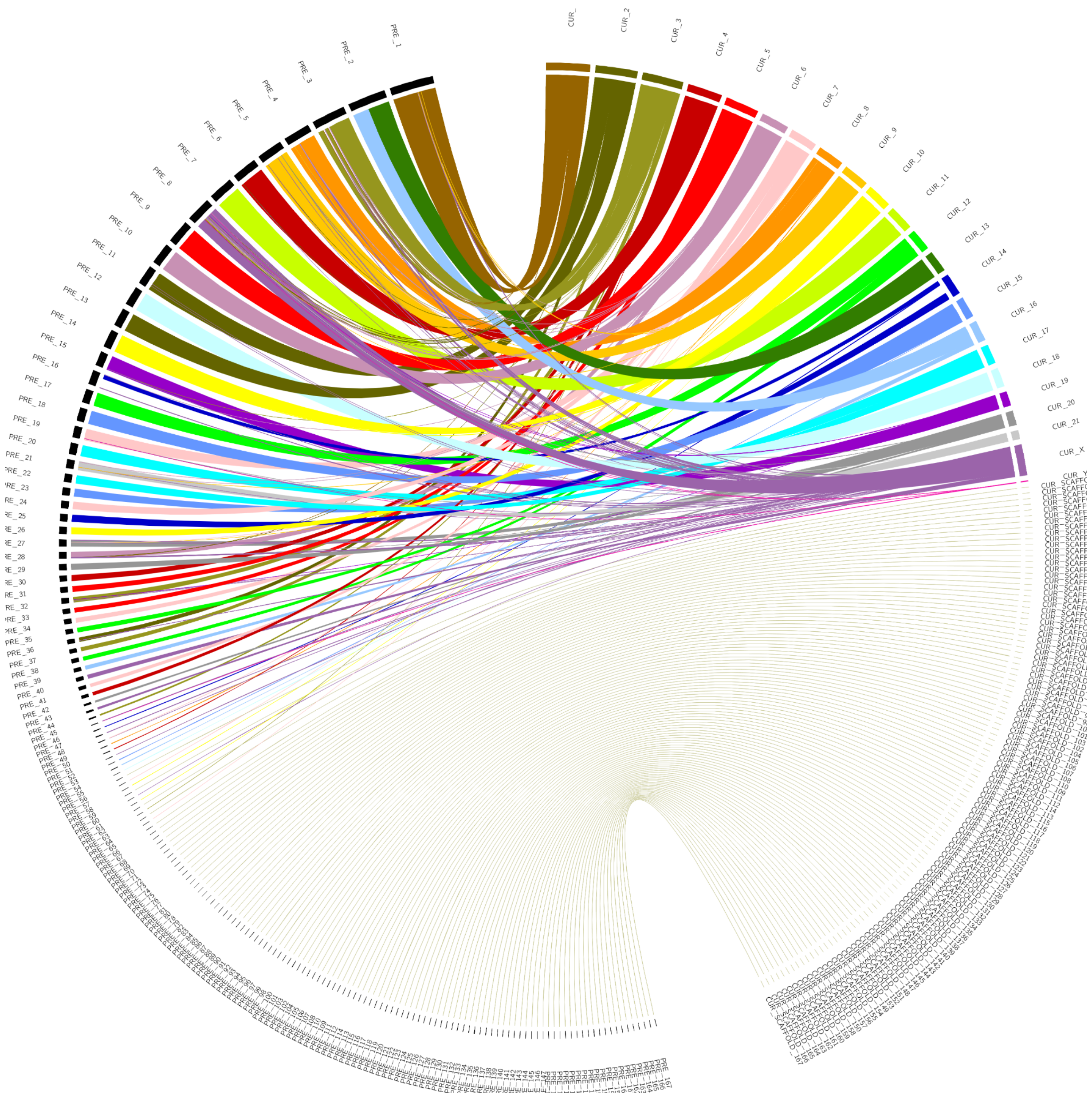

Supplementary Figure S1. Manual curation

A. Dot plot showing blue whale genome assembly before and after curation on vertical and horizontal axis respectively

B. Circos plot showing blue whale genome assembly before curation on the left and after curation on the right

Partial assembly of the *Y* chromosome

The blue whale whose genome is reported here is a male. However, assembling mammalian *Y* chromosomes is challenging due to their high repeat content and the presence of a large region of homology to the *X* chromosome, known as the pseudoautosomal region or *PAR*. We were able to identify a large, 132 MB scaffold, super-scaffold 8, as the *X* chromosome. Consistent with having a single copy of the *X* chromosome, this super-scaffold had two-fold lower coverage than most of the assembly throughout most of its length. It also had a region of normal coverage near the 5' end, corresponding to the *PAR* (Supplementary Figure S2.A). The *PAR* tends to have normal coverage because reads from both *X* and *Y* chromosomes map to this region. Additionally, super-scaffold 8 was homologous to the human *X* chromosome throughout most of its length (Supplementary Figure S2.B).

Karyotyping indicated that the *Y* chromosome was, as expected, only a fraction of *X* in size (Supplementary Figure S2.C). We were initially unable to annotate it due to our inability to find a high-confidence match to the *SRY* gene, a widely used mammalian *Y* chromosome marker. *SRY* was, however, identified in our alternative, 10X-based assembly, where it is located on a 11.3 MB scaffold that also encompasses the *PAR*, as shown in Supplementary Figure S2.D-E. In the final version of our assembly, we split off a ~2.4 MB region off the 5' end of super-scaffold 8 and designated it super-scaffold 23, a partial chromosome *Y*. Supplementary Figure S3 shows the relevant chromatin interaction maps computed from our Hi-C data.

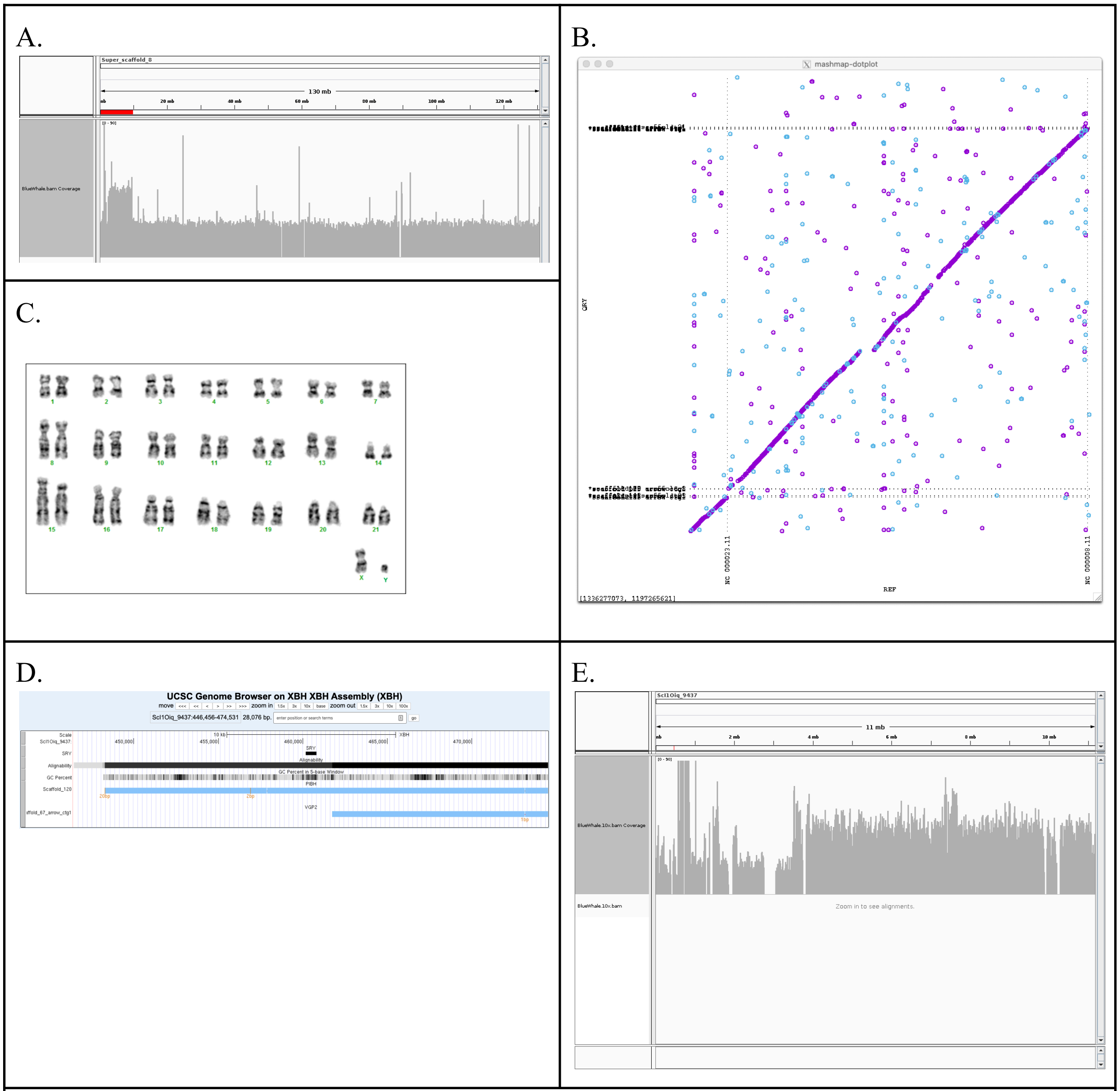

Supplementary Figure S2. Sex chromosomes.

A. Blue whale super-scaffold 8 coverage plot in IGV. The scaffold is shown before a ~2.4 MB segment was split off from the 5’ end to be designated as super-scaffold 23, the putative partial *Y* chromosome. A region thought to correspond to the *PAR* and the *Y* chromosome is highlighted. This region aligns to scaffold 9437 in the 10X-based assembly

B. MashMap dot plot shows that blue whale super-scaffold 8 is homologous to human *X* chromosome

C. Blue whale karyotype

D. Cactus alignment of 10X-based (XBH2), alternative PacBio-based (PIBH), and VGP assemblies in the region that contains *SRY*. XBH2 serves as the reference

E.	10X-based (XBH2) assembly scaffold 9437 coverage plot in IGV. The *SRY* gene is marked in red. The higher-coverage region on the right, between 3.5 and 11 Mbp, is *PAR*

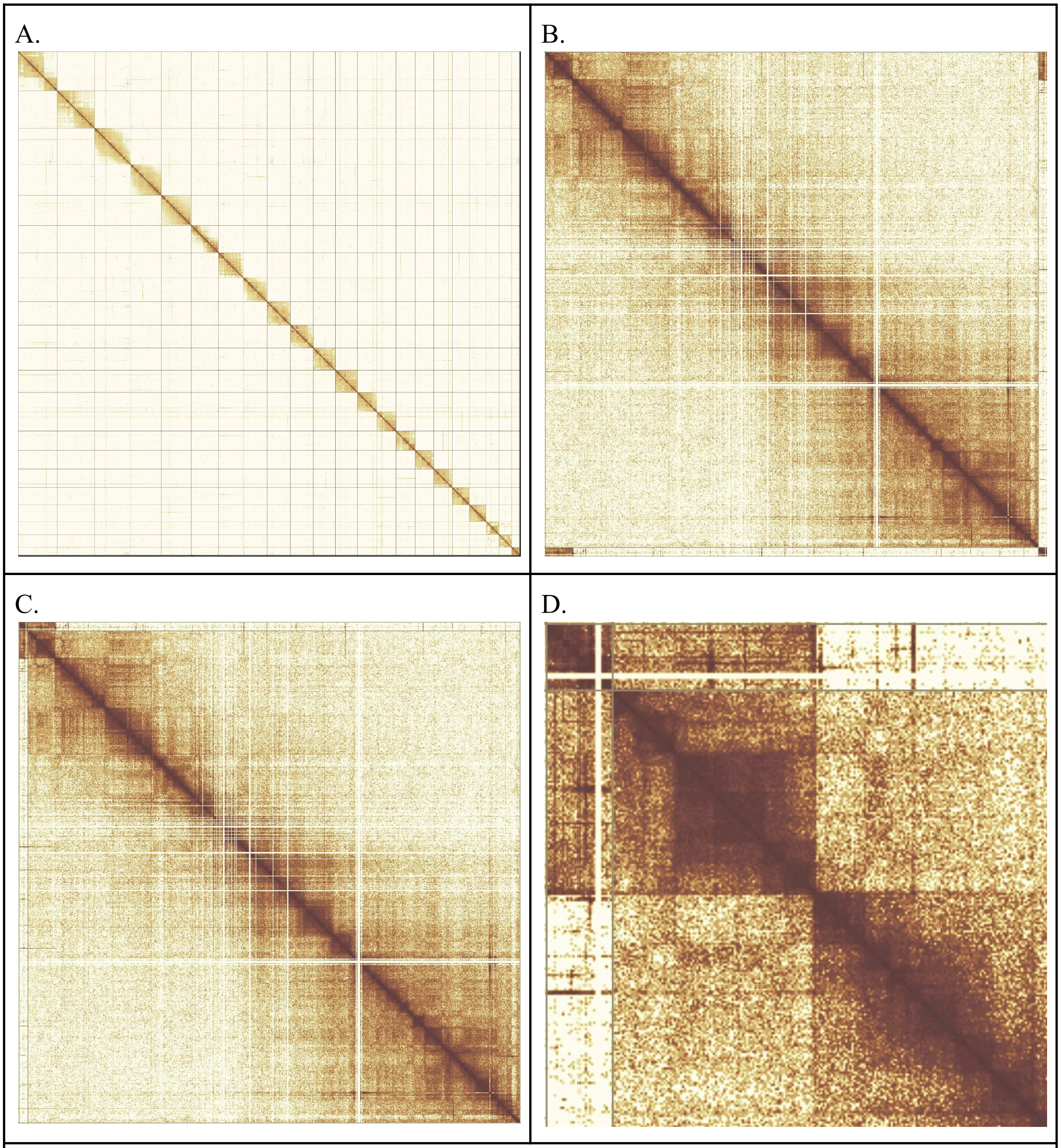

Supplementary Figure S3. Hi-C contact maps and the sex chromosomes.

A. A whole-genome contact map. The *X* chromosome is the 6'th from the top-left. The partial *Y* chromosome is too small to be clearly discernible on this map. However, it occupies the bottom-right corner of the *X* and was

initially assigned to the same scaffold.

B. The same map zoomed in to the *Y* chromosome and *PAR*. The partial *Y* chromosome is in the bottom-right corner. *PAR* is at top-left. An off-diagonal block of interactions between the partial *Y* and the *PAR* can be observed at bottom-left and top-right.

C. Same as (B), but with the partial *Y* moved to the top-left corner of the plot, so as to locate it next to the *PAR*.

D. Zoom-in on the top-left corner of (C) to show the partial *Y* and the *PAR* more clearly.

Segmental duplications

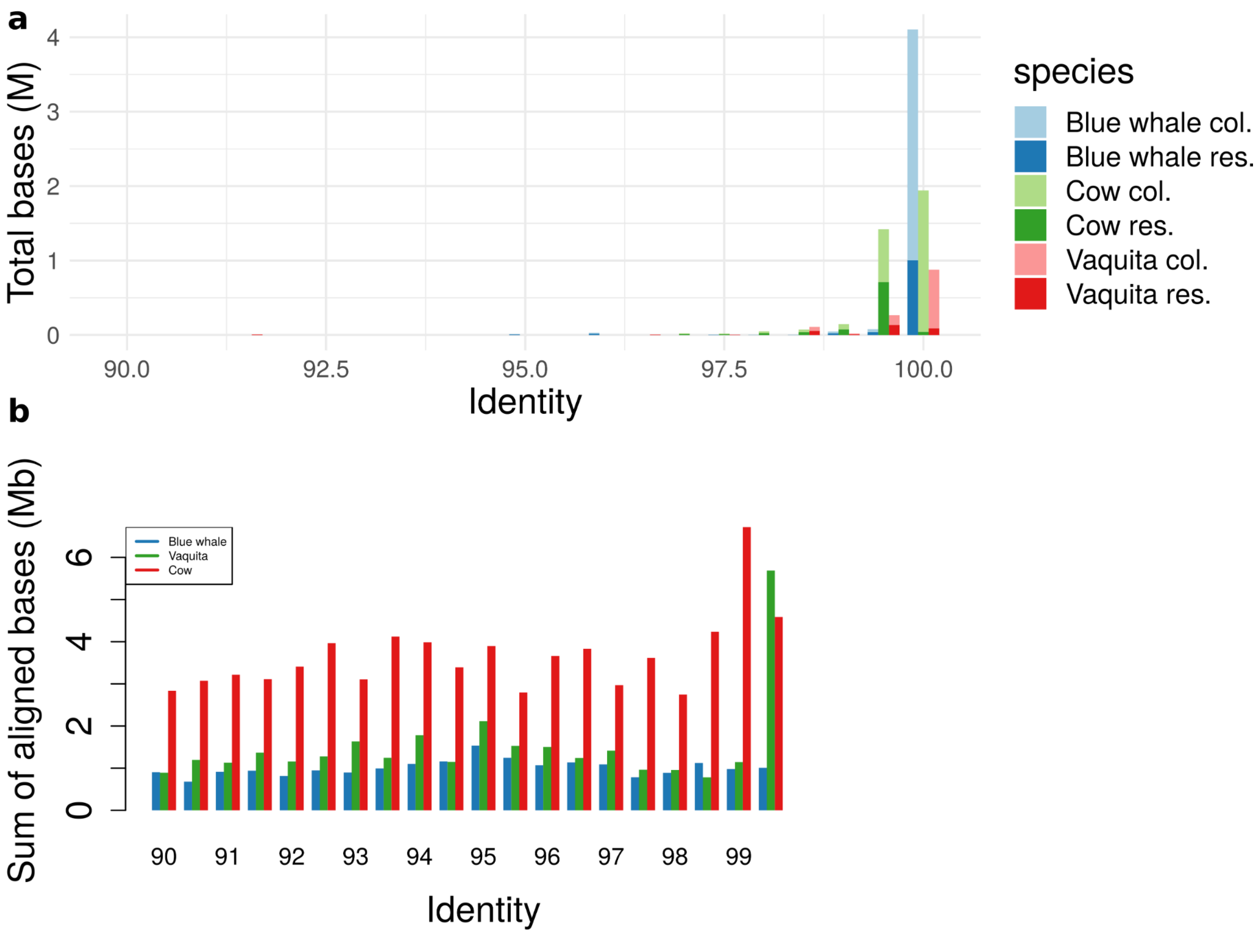

Supplementary Figure S4. Segmental duplications. The segmental duplications were measured by the total number of duplicated bases overlapping coding sequences (a) and according to the segmental duplication detection software sedef (b). When considering resolved duplications (b), there is an increase in the number of duplications in recent history in both cow and vaquita. However, when considering duplicated genes that are collapsed in any assembly and inferred by read depth, there is a roughly two-fold increase in the number of duplicated bases in blue

whale compared to vaquita, and four-fold compared to cow. This indicates a large recent burst in duplicated genes in blue whale, even if they are not entirely resolved in the assembly.

XRCC1 read coverage

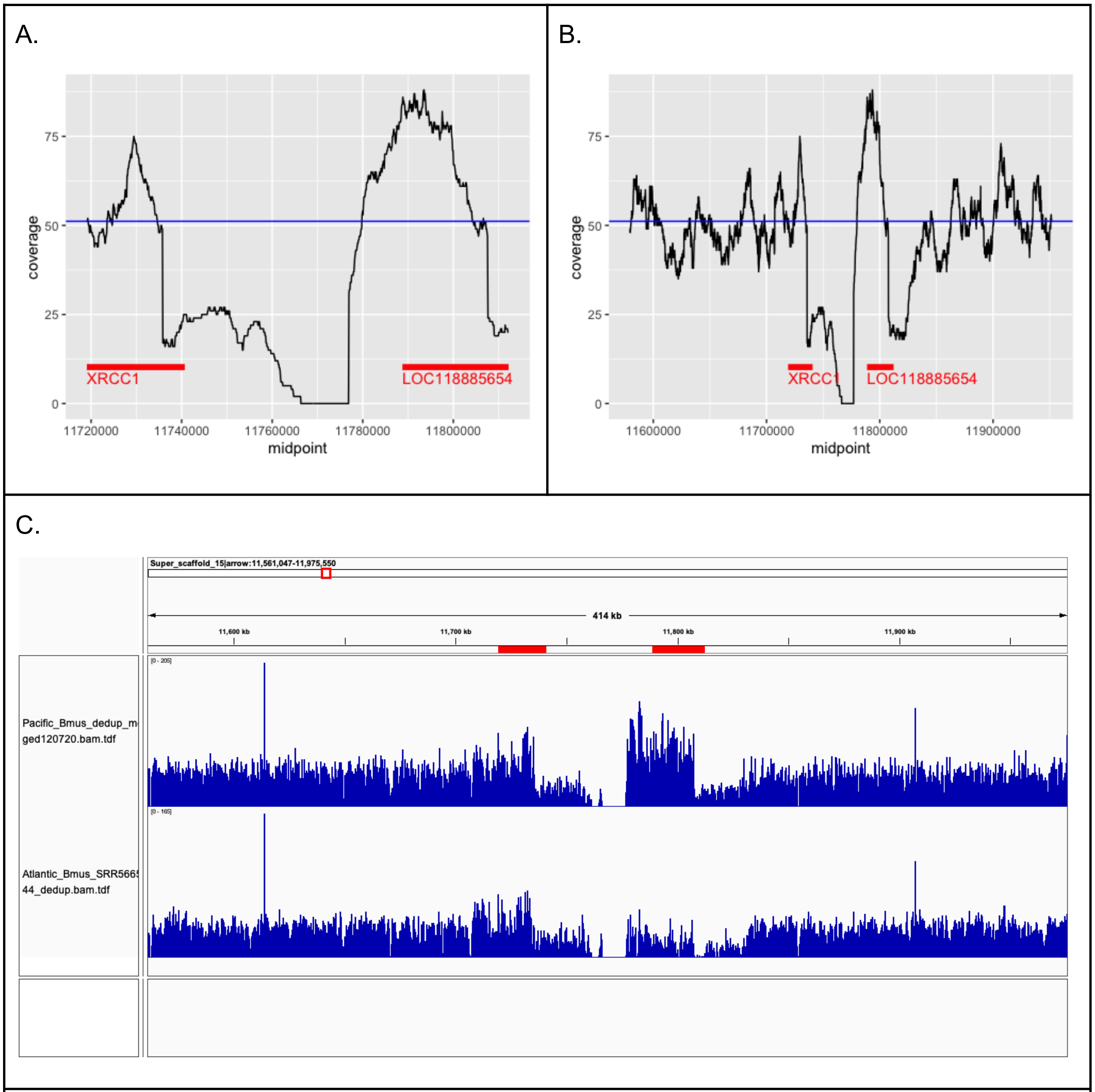

Supplementary Figure S5. XRCC1 read coverage. A. Binned PacBio coverage data. **B.** Same as A, zoomed-out. **C.** Illumina coverage data in the Pacific blue whale from this study (top) and Atlantic from (Árnason et al. 2018) (bottom), computed using igvtools and visualized in IGV (Robinson et al. 2011)

Synteny dot plots of CDK20 and CHRNB1 in blue whale vs. vaquita

A.

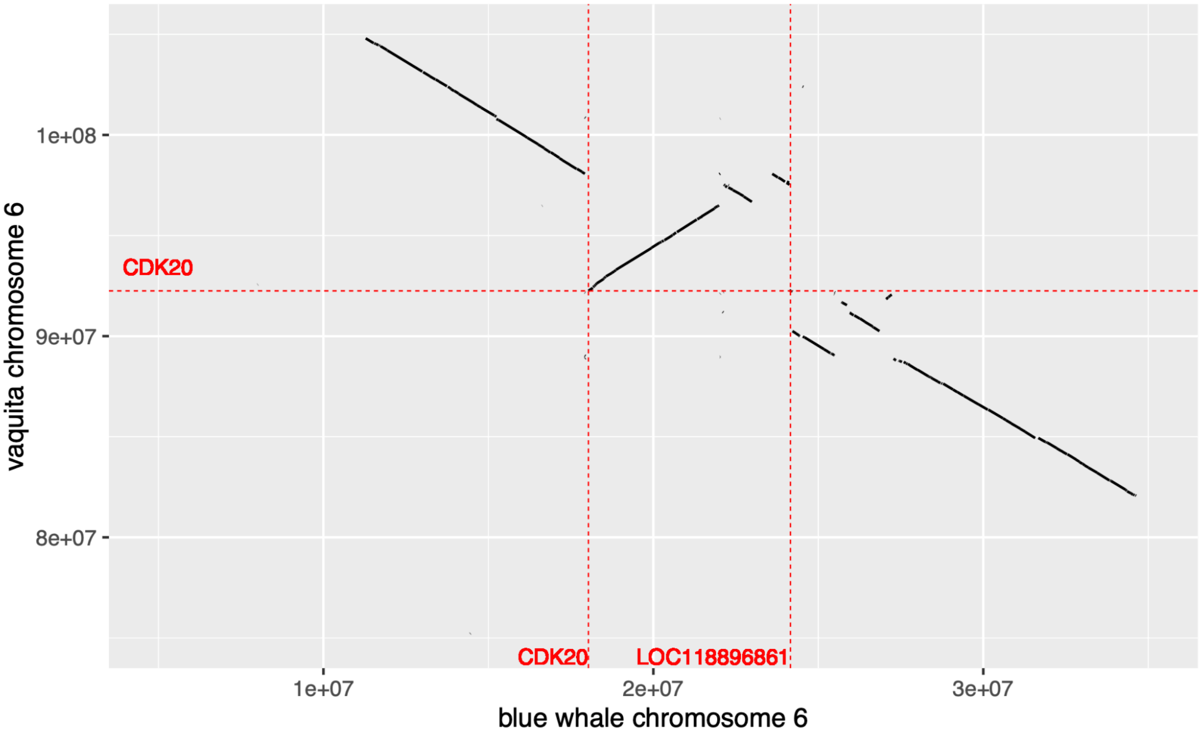
CDK20
1e+08
9e+07
8e+07
vaquita chromosome 6
CDK20
LOC118896861
1e+07
2e+07
3e+07
blue whale chromosome 6

B.

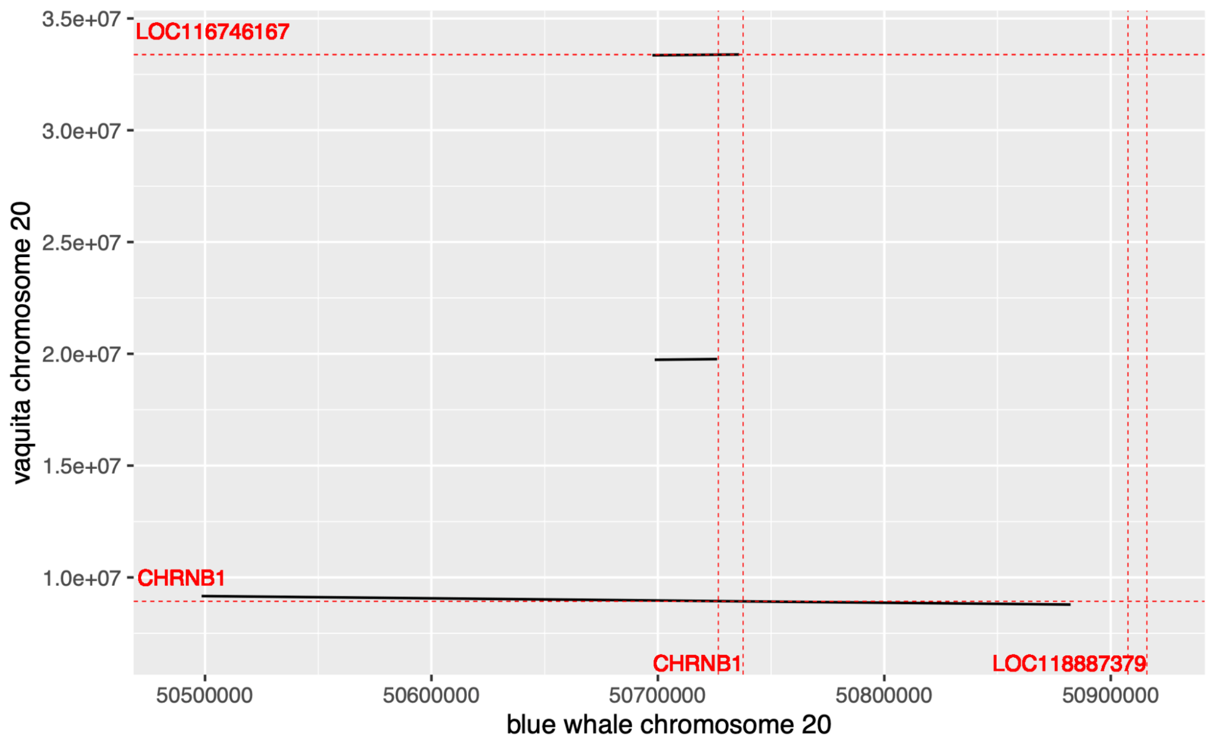

Supplementary Figure S6. Synteny dot plots of example genes. **A**. A synteny plot of the genomic regions containing *CDK20* in blue whale and vaquita. Locations of *CDK20* genes are marked by dashed red lines. **B**. A synteny plot of genomic regions containing *CHRNB1* in blue whale and vaquita. 5′ and 3′ ends of *CHRNB1* genes are marked by dashed red lines. The starts and ends of the two genes can be distinguished in blue whale, but not vaquita, due to the two copies' close proximity in the former and a large distance between them in the latter. While both gene copies in the blue whale are intact, the second copy in vaquita is a pseudogene containing several deactivating mutations (not shown).

Population history supplementary figures

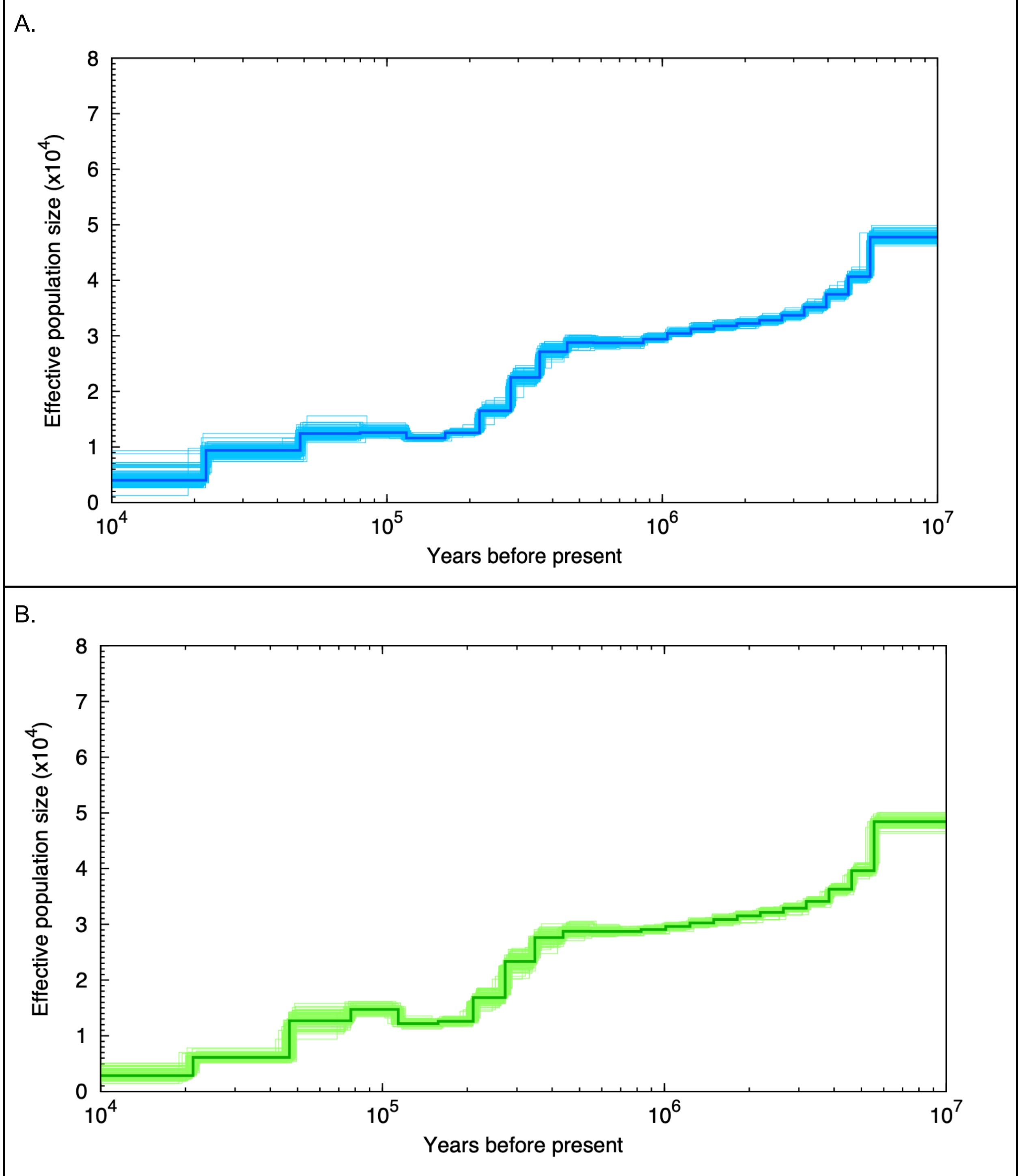

Supplementary Figure S7. Individual PSMC plots for Pacific and Atlantic blue whales. Short read data mapped to the reference genome, and repeats masked. 100 bootstrapped plots included. The generation time for blue whale (30.8 years) was used with the average substitution rate for mysticetes (1.58E-08

substitutions/site/generation; Jackson et al., 2009). **A**. Pacific (PSMC atomic intervals: all intervals >10). **B**. Atlantic (PSMC atomic intervals: intervals 62-63 <10 (7.4, 5.9))

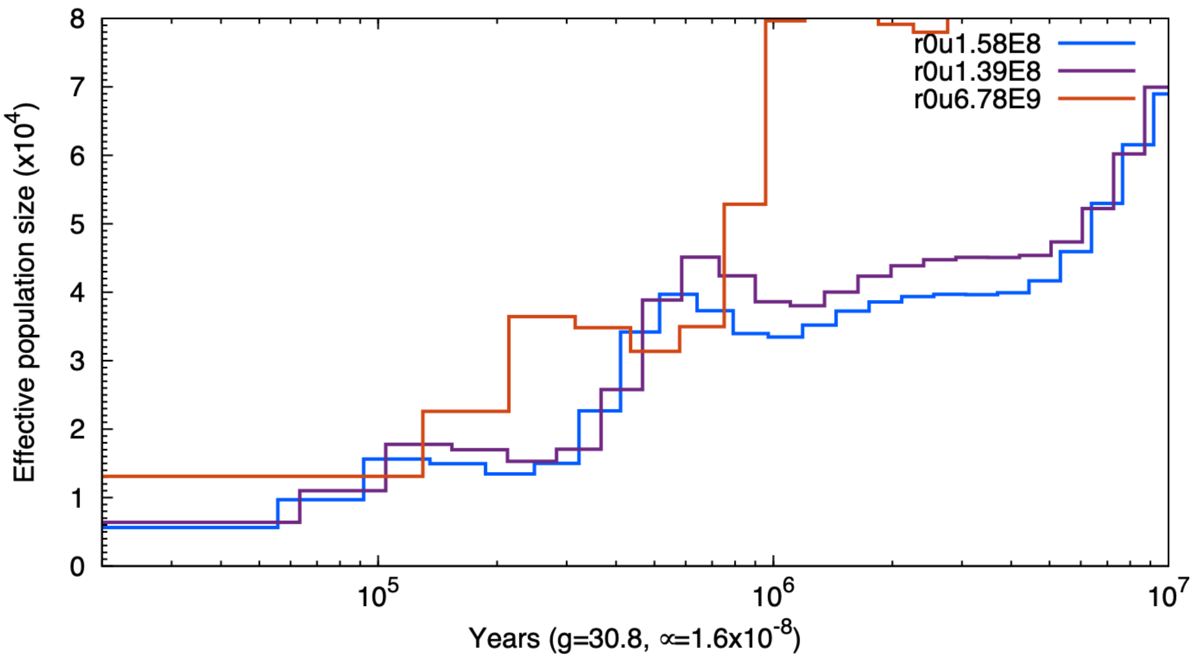

Supplementary Figure S8. Effect of different mutation rates on plot of PSMC results. The mutation rates used were the mean value for mysticetes from (Jackson et al. 2009) (1.5878E-08 substitutions/site/generation); the lowest rate from (Jackson et al. 2009), used by (Árnason et al. 2018) (1.3978E-08 substitutions/site/generation); and the mean rate for mysticetes as determined by (Dornburg et al. 2012) (6.78E-09 substitutions/site/generation). The slower mutation rates have the result of shifting the plot to the right (older) and up (larger population sizes).

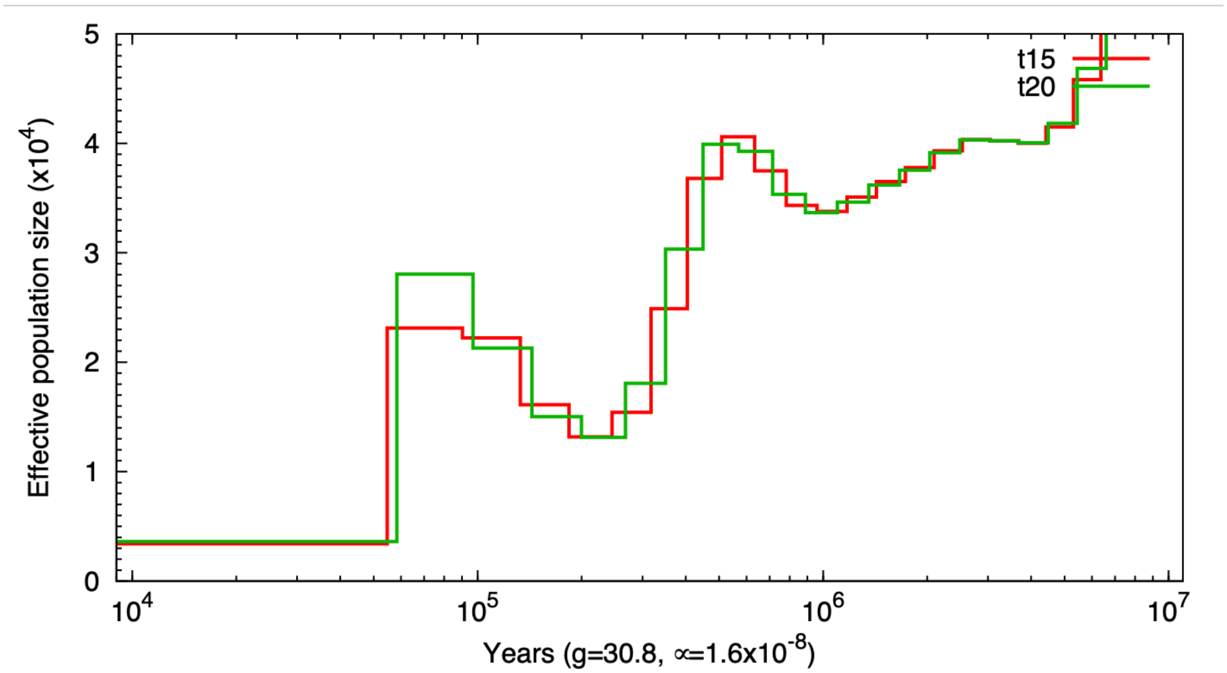

Supplementary Figure S9. Effect of different maximum coalescent times (15 vs. 20) on plot of PSMC results (for Atlantic blue whale).

Supplementary Methods

Blue whale sample acquisition

The blue whale skin sample was obtained from a photographically identified free ranging individual off the coast of Santa Barbara, CA, using standard cetacean biopsy techniques (Lambertsen 1987). A custom stainless-steel biopsy tube (8 mm OD x 38 mm long) was fitted into a 25mm OD stopper at the end of a crossbow arrow. Tissue was retained by three dental broaches set in the center of the base (a modification of (Barrett-Lennard et al. 1996)). The biopsy sample was extracted from the tip of the biopsy tube in the field and immediately transferred into a Falcon tube containing ice-cold whale biopsy medium composed of Alpha-MEM (Corning), 5% fetal bovine serum (Millipore), and 2x Antibiotic-Antimitotic (Life Technologies) and kept on ice until processing for cell culture.

Fibroblast cell culture

The skin biopsy sample was dissected into epidermis, dermis, and blubber tissue. The dermis section was cut into small pieces and digested for 30 min at 37ºC in 1ml of a 0.125 mg/ml solution of Liberase TM (Roche). 5 ml of pre-warmed fibroblast medium composed of a 50:50 mix of Alpha-MEM (Corning), 10% fetal bovine serum (Millipore) with 1x Antibiotic-Antimitotic (Life Technologies) and FBM complete (LONZA) was added to the digested tissue pieces and the suspension was transferred to two gelatin-coated T25 tissue culture flasks (Corning). Spent medium was replaced carefully every other day without disturbing the adhering tissue pieces. After 2 weeks of culture at 33ºC, 4% CO_2, 5% O2 the primary blue whale fibroblasts were passaged, banked, and expanded for Genomic DNA isolation.

Karyotyping

The Blue Whale karyotype was obtained from cultured fibroblasts by G-banding (Houck et al. 2017).

HMW DNA prep

A genomic DNA-seq High Molecular Weight (HMW) sample was processed from twenty, 10 cm plates containing Blue Whale fibroblast cells, BW-04. Briefly, HMW gDNA was purified from the Gentra Puregene Cell Isolation Kit (Qiagen, Netherlands). The sample was quantified using the PicoGreen Assay (Thermofisher, Massachusetts) and equaled 250 micrograms of HMW Blue Whale gDNA. Gender was determined using the Amel SC primer set in a PCR reaction and run on a 1.2% agarose gel (Macé and Crouau-Roy 2008). Sample BW-04, was confirmed to be male. Gender was determined using the Amel SC primer set in a PCR reaction and run on a 1.2% agarose gel (Macé and Crouau-Roy 2008). Sample BW-04, was confirmed to be male.

Illumina gDNA-seq

Illumina genomic DNA sequencing data used to generate the base-level accuracy metrics reported in Table 1 was generated as follows. DNA-seq samples were processed from Blue Whale fibroblast cells, BW-04. Briefly, DNA was purified from the Blood and Tissue DNA Isolation Kit (Qiagen, Netherlands). The Nextera DNA Flex Library Prep Kit (Illumina, San Diego) was used to prepare and index all gDNA libraries. Libraries were cleaned and size selected using the Select-a-Size DNA Clean and Concentrator Kit (Zymo Research, Irvine). Blue whale fibroblast cell libraries were pooled and run on two Illumina HiSeq 3000 lanes with

paired-end 151 base pair reads and 8 base pair dual index reads. Read data were generated by using Illumina's CASAVA-1.8.2 base calling software to demultiplex samples. Reads were then filtered to remove adapters and low-quality bases on the ends.

Optical mapping

High Molecular Weight DNA was extracted from frozen cells with the Bionano Prep™ Frozen Cell Pellet DNA Isolation Kit. The long DNA molecules were labeled with the DLE-1 enzyme according to the Bionano Prep Direct Label and Stain protocol. The labeled molecules were loaded into a single flowcell of a Saphyr Chip, and the chip was run on the Saphyr Optical Mapping Instrument, collecting the images of 439.46 Gb molecules greater than 150kb with minimum of 9 labels.

Hi-C

A Dovetail Hi-C library was prepared in a similar manner as described previously (Lieberman-Aiden et al. 2009). Briefly, for each library, chromatin was fixed in place with formaldehyde in the nucleus and then extracted Fixed chromatin was digested with DpnII, the 5′ overhangs filled in with biotinylated nucleotides, and then free blunt ends were ligated. After ligation, crosslinks were reversed and the DNA purified from protein. Purified DNA was treated to remove biotin that was not internal to ligated fragments. The DNA was then sheared to ~350 bp mean fragment size and sequencing libraries were generated using NEBNext Ultra enzymes and Illumina-compatible adapters. Biotin-containing fragments were isolated using streptavidin beads before PCR enrichment of each library. The libraries were sequenced on an Illumina HiSeq X to produce 402 million 2x150 bp paired end reads, which provided 23,471.34 x physical coverage of the genome (10-10,000 kb pairs).

Illumina RNA-seq

RNA-seq samples were processed from Blue Whale fibroblast cells, BW-04. Total RNA was purified from RLT-Plus Buffer using RNeasy Plus Mini Kits (Qiagen, Netherlands). The TruSeq RNA Library Prep Kit v2 (Illumina, San Diego), as per the manufacturer's directions, was used to prepare and index all cDNA libraries. Three uniquely indexed samples were pooled together in one HiSeq 3000 lane with paired end 150 base pair reads and 8 base pair dual index reads. RNA-seq reads were generated by using CASAVA-1.8.2, filtering for quality and adapters. The

RNA data was submitted to the SRA public archives, under accession # SRX7696402, and used for annotation of the genome.

Iso-seq

We prepared PacBio Iso-seq libraries according to the "Procedure & Checklist -Iso-Seq™ Template Preparation for Sequel Systems" (PN 101-070-200 version 05). cDNA was transcribed using the SMRTer PCR cDNA synthesis kit (Clontech, Mountain View, CA) from 300ng of total RNA. Once amplified, the cDNA was cleaned with ProNex beads prior to PacBio library preparation. We used Pacific Biosciences SMRTbell® Express Template Prep Kit 2.0 (#100-938-900) following the manufacturer protocol to ligate SMRTbell PacBio adapters. The final PacBio Iso-seq library was sequenced on one PacBio 1M SMRT Cell (101-500-400) on the Sequel instrument with the binding kit 3.0 (101-500-400) and the sequencing kit 3.0 (PN 101-746-800) using the Sequel II Binding Kit 1.0 (101-427-500) and 10 hr movie with fours hours extension. 20.2Gb of raw data was generated with an average insert length of 3,165bp and an average polymerase read length of 40,317bp. CCS were generated and full length cDNA identified and clustered into 20,665 high-quality isoforms using the Isoseq3 workflow in smrtlink 6.0.0

10X Genomics sequencing

A genomic DNA-seq High Molecular Weight (HMW) sample was prepared as described above. The 10X Genomics Chromium library was prepared by the University of Minnesota Genomics Center (Minneapolis, MN) following the protocol for the Genome Reagent Kit v2 chemistry (10X Genomics, cat. 120258) and modified accordingly for a 3.0 Gb genome. In brief, genomic DNA quality was visualized via pulsed field gel electrophoresis on a CHEF-DR II System (Bio-Rad, cat.1703725) and Genomic DNA ScreenTape (Agilent 2200 TapeStation, cat. 5067-5365) to verify an average fragment DNA length of > 50 Kb. DNA was diluted without size selection to within the target range for a 3.0 Gb genome and the resulting concentration was validated in triplicate by Qubit Fluorometric Quantitation (Life Technologies, cat. Q32854). Using the 10X Chromium Controller, Gel Bead-in-Emulsions (GEMS) were generated by loading the DNA onto the microfluidic Chromium Genome Chip (10X Genomics, cat. 120257) with partitioning oil and a library of Genome Gel Beads to add a specific 16-bp barcode. Library construction was completed by incorporating P5 and P7 primers, Read 2, and an i7 sample index

(10X Genomics, cat. 120262) via an 8-cycle library amplification. The fragment size of the library was analyzed using the High Sensitivity DNA Assay (Agilent BioAnalyzer, cat. 5067-4626) and quantified using KAPA Library Quantification Kit for Illumina platforms (Kapa Biosystems, cat. KK4873).

The University of Minnesota prepared four HMW gDNA Blue Whale samples with four unique 10X indexes from the original BW-04 stock sample, pooled the four indexed gDNA libraries into one tube, and shipped these 10X gDNA BW-04 libraries back to the Morgridge Institute DNA Sequencing Team. Blue whale HMW gDNA 10X pooled libraries were then run on three Illumina HiSeq 3000 lanes with paired-end 170 base pair reads and 8 base pair single index read.

10X-based genome assembly workflow

We generated an initial assembly based on Illumina sequencing of a library prepared using 10X Genomics to produce synthetic long reads (see Methods, 10X Genomics sequencing). The data were assembled using 10X Supernova software version 2.0.1 (Weisenfeld et al. 2017), generating two pseudohaplotypes using the following commands:

```
supernova mkfastq
--run=/illumina_runs/180523_J00168_0045_AHNY5VBBXX --csv
layout.csv --use-bases-mask=y170,i8,y170 --delete-undetermined
--output-dir=180523_J00168_0045_AHNY5VBBXX_fastq --localcores=95
--localmem=6000 |& tee superova_mkfastq.out

supernova run --id=Blue_Whale_4_10X_Supernova_run1
--fastqs=180523_J00168_0045_AHNY5VBBXX_fastq --localcores=95
--localmem=5500 |& tee Blue_Whale_4_10X_Supernova_run1.out

supernova mkoutput --style=pseudohap2 --asmdir=../outs/assembly/
--outprefix=ybukhman_Blue_Whale_4_10X_Supernova_run1_pseudohap2 |&
tee
ybukhman_Blue_Whale_4_10X_Supernova_run1_mkoutput_pseudohap2.out
```

The first pseudohaplotype was further scaffolded as follows. Scaffolding using optical maps was performed by McDonnell Genome Institute (MGI) at the Washington University in St. Louis. Optical mapping data were assembled using Bionano Solve v.3.2.1 software and optArguments_nonhaplotype_noES_DLE1_saphyr.xml parameters into 742 genome maps with an N50 of 36.72Mb and a total length of 2779.23Mb. Hybrid scaffolding between the bionano genome maps and the sequence contigs was performed with default hybridScaffold_DLE1_config parameters, resolving conflicts in both the optical map and sequence assemblies.

Additional scaffolding using Hi-C data was performed by Dovetail Genomics. The input *de novo* assembly, shotgun reads, and Dovetail Hi-C library reads were used as input data for HiRise, a software pipeline designed specifically for using proximity ligation data to scaffold genome assemblies (Putnam et al. 2016). Shotgun and Dovetail Hi-C library sequences were aligned to the draft input assembly using a modified SNAP read mapper (Zaharia et al. 2011). The separations of Dovetail Hi-C read pairs mapped within draft scaffolds were analyzed by HiRise to produce a likelihood model for genomic distance between read pairs, and the model was used to identify and break putative misjoins, to score prospective joins, and make joins above a threshold. After scaffolding, shotgun sequences were used to close gaps between contigs. Short scaffolds were filtered out using PRINSEQ software on the GenSAS web site (Schmieder and Edwards 2011; Humann et al. 2019). We refer to the final version of this assembly as *XBH2*.

Mitogenome assembly

We have assembled the blue whale mitogenome using the mitoVGP pipeline (Formenti et al. 2021; Rhie et al. 2021). This assembly is highly consistent with the Genbank reference sequence for the blue whale (MF409242.1). The two sequences have the same length (16,403 bp) with 99.76% identity, and no repeated motifs of significant length or duplicated genes are present. Differences are single or dinucleotides and can therefore be interpreted as individual SNPs. Mitos2 annotation of the mitogenome assembly generated no significant warning. The genes most affected by SNPs include the first ribosomal RNA gene (rrnS), nad2, nad5 and the control region.

Additional false duplication removal and polishing steps

We found that the primary assembly still contained spurious false duplications and an initial attempt to annotate it by NCBI GNOMON workflow found potential frameshift errors in 13% of its protein coding genes (National Center for Biotechnology Information (US) 2017; Formenti et al. 2022). Therefore, both primary and alternate pseudohaplotype assemblies were once again purged of false duplicates using purge_dups a more advanced software than purge-haplotigs for removing false duplications within contigs (Guan et al. 2020), and re-polished using Arrow with an optimized coverage cut-off parameter of 31. This removed approximately 1 Mb of sequence from the primary assembly and 40 Mb from the alternate and lowered the frequency of genes with frameshift errors to an acceptable level of 10%.

Prediction of GO terms

We predicted Gene Ontology (GO) terms with Phylo-PFP (Jain and Kihara 2019), using protein sequences inferred by the NCBI annotation workflow and the November 2019 version of the Phylo-PFP database. Phylo-PFP is a sequence-based protein function prediction method, which mines functional information from a broad range of similar sequences, including those with a low sequence similarity identified by a PSI-BLAST search. The sequences retrieved from PSI-BLAST are reranked by considering the phylogenetic distance and the sequence similarity to the query. Incorporating phylogenetic information leads to better functional similarity estimation. GO terms of each retrieved protein are assigned the same score as the sequence. Finally, for each GO term, scores from all sequences are summed. The prediction is also enriched with GO terms that have greater than 90% probability of co-occurrence.

TOGA application

We applied TOGA using the human GRChg38 assembly and the human GENCODE V38 gene annotation as reference to genomes of cetaceans (query species) (Kirilenko et al. 2022). Briefly, TOGA uses pairwise genome alignment chains and machine learning to infer orthologous loci for each transcript in the reference annotation, utilizing that orthologous genes exhibit alignments in intronic and flanking intergenic regions. For each orthologous locus, TOGA projects the reference transcript to its orthologous query locus using CESAR 2.0 (Sharma et al. 2017), resulting in an annotated transcript in the query genome. For each projected transcript, TOGA determines whether it encodes an intact reading frame, has gene-inactivating mutations or missing sequence caused by assembly gaps.

ROH analysis

ROH were identified in a gVCF file with loci called using a pipeline available on github (Wolf 2023). In brief, Illumina short reads were trimmed for quality and adapter sequences using FASTP v0.23.2 (Chen et al. 2018) with the options "-g -3 -l 40 -y -c -p". Trimmed reads were mapped to the repeat-masked assemblies of both the final long-read assembly and the assembly based on linked short reads (10X Genomics). Mapping was performed using BWA MEM v0.7.17-r1188 (http://bio-bwa.sourceforge.net) and SAMTOOLS v1.9 sort (Danecek et al. 2021) (Danecek et al. 2021) using default settings. Potential duplicates were removed and read-groups were added using the PICARD v2.21.2-0 toolkit (https://broadinstitute.github.io/picard/). Genotype-calling was done per BAM file using BCFTOOLS v1.12 *mpileup* and BCFTOOLS

v1.12 *call* (Danecek et al. 2021) with the “-c” flag and minimum mapping- and base-quality cutoffs of 20 and 13, respectively. All inferred sites were further filtered by excluding sites with divergent read coverage (>3-fold and <0.3-fold of the expected individual mean coverage) and sites with more than 25% missing data using the BCFTOOLS filter function.

To identify ROH, the DARWINDOW (de Jong 2021) pipeline was used to scan through the loci previously called. DARWINDOW uses a non-overlapping sliding window approach to calculate heterozygosity per window and merges homozygous windows to ROHs afterwards. This process is dependent on window size, heterozygosity threshold, and minimal number of windows per ROH. Therefore, DARWINDOW visualizes the heterozygosity distribution and identifies ROHs per scaffold in line-charts (Figure 8C) allowing for manually adjusting these settings until ROH identification calls all visible ROHs correctly. Doing so, it was found that a window size of 20 Kb, a minimal window number of 25 and a heterozygosity threshold of 2.5% works best for the 23 Super-scaffolds of the long-read assembly. Using these settings, the analysis was repeated for the linked-short-read assembly and then compared to the long-read assembly by sorting ROHs in bins depending on their length (Figure 8 A+B). Inbreeding coefficients (FROH) were calculated as the proportion of these ROHs compared to the total length of all sites in the respective assembly.

R session info

```
> sessionInfo()
R version 4.1.0 (2021-05-18)
Platform: x86_64-apple-darwin17.0 (64-bit)
Running under: macOS Big Sur 10.16

Matrix products: default
LAPACK:
/Library/Frameworks/R.framework/Versions/4.1/Resources/lib/libRlapack.dylib

locale:
[1] en_US.UTF-8/en_US.UTF-8/en_US.UTF-8/C/en_US.UTF-8/en_US.UTF-8

attached base packages:
[1] parallel  stats4    stats     graphics  grDevices utils     datasets
methods   base
```

```
other attached packages:
 [1] optparse_1.7.1                   sequencing_1.16.0
 [3] RNAseqData.HNRNPC.bam.chr14_0.30.0 BSgenome.Hsapiens.UCSC.hg19_1.4.3
 [5] BSgenome_1.60.0                  AnnotationHub_3.0.1
 [7] BiocFileCache_2.0.0              dbplyr_2.1.1
 [9] VariantAnnotation_1.38.0         rtracklayer_1.52.0
[11] ShortRead_1.50.0                 BiocParallel_1.26.0
[13] GenomicAlignments_1.28.0         Rsamtools_2.8.0
[15] Biostrings_2.60.1                XVector_0.32.0
[17] SummarizedExperiment_1.22.0      Biobase_2.52.0
[19] MatrixGenerics_1.4.0             matrixStats_0.59.0
[21] GenomicRanges_1.44.0             GenomeInfoDb_1.28.0
[23] IRanges_2.26.0                   S4Vectors_0.30.0
[25] BiocGenerics_0.38.0              rentrez_1.2.3
[27] forcats_0.5.1                    stringr_1.4.0
[29] dplyr_1.0.7                      purrr_0.3.4
[31] readr_1.4.0                      tidyr_1.1.3
[33] tibble_3.1.2                     ggplot2_3.3.4
[35] tidyverse_1.3.1

loaded via a namespace (and not attached):
 [1] colorspace_2.0-1           rjson_0.2.20               hwriter_1.3.2
 [4] ellipsis_0.3.2             fs_1.5.0
rstudioapi_0.13
 [7] getopt_1.20.3              bit64_4.0.5
interactiveDisplayBase_1.30.0
[10] AnnotationDbi_1.54.1       fansi_0.5.0
lubridate_1.7.10
[13] xml2_1.3.2                 cachem_1.0.5               jsonlite_1.7.2
[16] broom_0.7.7                png_0.1-7                  shiny_1.6.0
[19] BiocManager_1.30.16        compiler_4.1.0             httr_1.4.2
[22] backports_1.2.1            assertthat_0.2.1           Matrix_1.3-3
[25] fastmap_1.1.0              cli_2.5.0                  later_1.2.0
[28] htmltools_0.5.1.1          prettyunits_1.1.1          tools_4.1.0
[31] gtable_0.3.0               glue_1.4.2
GenomeInfoDbData_1.2.6
[34] rappdirs_0.3.3             tinytex_0.32               Rcpp_1.0.7
[37] cellranger_1.1.0           vctrs_0.3.8                xfun_0.24
```

```
[40] rvest_1.0.0                 mime_0.10
lifecycle_1.0.0
[43] restfulr_0.0.13             XML_3.99-0.6
zlibbioc_1.38.0
[46] scales_1.1.1                promises_1.2.0.1           hms_1.1.0
[49] RColorBrewer_1.1-2          yaml_2.2.1                 curl_4.3.1
[52] memoise_2.0.0               biomaRt_2.48.1
latticeExtra_0.6-29
[55] stringi_1.6.2               RSQLite_2.2.7
BiocVersion_3.13.1
[58] BiocIO_1.2.0                GenomicFeatures_1.44.0     filelock_1.0.2
[61] rlang_0.4.11                pkgconfig_2.0.3            bitops_1.0-7
[64] lattice_0.20-44             bit_4.0.4
tidyselect_1.1.1
[67] magrittr_2.0.1              R6_2.5.0                   generics_0.1.0
[70] DelayedArray_0.18.0         DBI_1.1.1                  pillar_1.6.1
[73] haven_2.4.1                 withr_2.4.2
KEGGREST_1.32.0
[76] RCurl_1.98-1.3              modelr_0.1.8               crayon_1.4.1
[79] utf8_1.2.1                  jpeg_0.1-8.1               progress_1.2.2
[82] grid_4.1.0                  readxl_1.3.1               blob_1.2.1
[85] reprex_2.0.0                digest_0.6.27              xtable_1.8-4
[88] httpuv_1.6.1                munsell_0.5.0
```

References

Árnason Ú, Lammers F, Kumar V, Nilsson MA, Janke A. 2018. Whole-genome sequencing of the blue whale and other rorquals finds signatures for introgressive gene flow. *Sci. Adv.* 4:eaap9873.

Barrett-Lennard LG, Smith TG, Ellis GM. 1996. A Cetacean Biopsy System Using Lightweight Pneumatic Darts, and Its Effect on the Behavior of Killer Whales. *Mar. Mammal Sci.* 12:14–27.

Chen S, Zhou Y, Chen Y, Gu J. 2018. fastp: an ultra-fast all-in-one FASTQ preprocessor. *Bioinformatics* 34:i884–i890.

Danecek P, Bonfield JK, Liddle J, Marshall J, Ohan V, Pollard MO, Whitwham A, Keane T, McCarthy SA, Davies RM, et al. 2021. Twelve years of SAMtools and BCFtools. *GigaScience* 10:giab008.

Dornburg A, Brandley MC, McGowen MR, Near TJ. 2012. Relaxed Clocks and Inferences of Heterogeneous Patterns of Nucleotide Substitution and Divergence Time Estimates across Whales and Dolphins (Mammalia: Cetacea). *Mol. Biol. Evol.* 29:721–736.

Formenti G, Rhie A, Balacco J, Haase B, Mountcastle J, Fedrigo O, Brown S, Capodiferro MR, Al-Ajli FO, Ambrosini R, et al. 2021. Complete vertebrate mitogenomes reveal widespread repeats and gene duplications. *Genome Biol.* 22:120.
Formenti G, Rhie A, Walenz BP, Thibaud-Nissen F, Shafin K, Koren S, Myers EW, Jarvis ED, Phillippy AM. 2022. Merfin: improved variant filtering, assembly evaluation and polishing via k-mer validation. *Nat. Methods*:1–9.
Guan D, McCarthy SA, Wood J, Howe K, Wang Y, Durbin R. 2020. Identifying and removing haplotypic duplication in primary genome assemblies. *Bioinformatics* [Internet]. Available from: https://academic.oup.com/bioinformatics/advance-article/doi/10.1093/bioinformatics/btaa025/5714742
Houck ML, Lear TL, Charter SJ. 2017. Animal cytogenetics. In: The AGT Cytogenetics Laboratory Manual. John Wiley & Sons, Ltd. p. 1055–1102. Available from: https://www.onlinelibrary.wiley.com/doi/abs/10.1002/9781119061199.ch24
Humann JL, Lee T, Ficklin S, Main D. 2019. Structural and Functional Annotation of Eukaryotic Genomes with GenSAS. In: Kollmar M, editor. Gene Prediction: Methods and Protocols. Methods in Molecular Biology. New York, NY: Springer New York. p. 29–51. Available from: https://doi.org/10.1007/978-1-4939-9173-0_3
Jackson JA, Baker CS, Vant M, Steel DJ, Medrano-González L, Palumbi SR. 2009. Big and Slow: Phylogenetic Estimates of Molecular Evolution in Baleen Whales (Suborder Mysticeti). *Mol. Biol. Evol.* 26:2427–2440.
Jain A, Kihara D. 2019. Phylo-PFP: improved automated protein function prediction using phylogenetic distance of distantly related sequences. *Bioinformatics* 35:753–759.
de Jong MJ. 2021. Darwindow. Available from: https://github.com/mennodejong1986/Darwindow
Kirilenko BM, Munegowda C, Osipova E, Jebb D, Sharma V, Blumer M, Morales A, Ahmed A-W, Kontopoulos D-G, Hilgers L, et al. 2022. TOGA integrates gene annotation with orthology inference at scale.
Lambertsen RH. 1987. A Biopsy System for Large Whales and Its Use for Cytogenetics. *J. Mammal.* 68:443–445.
Lieberman-Aiden E, van Berkum NL, Williams L, Imakaev M, Ragoczy T, Telling A, Amit I, Lajoie BR, Sabo PJ, Dorschner MO, et al. 2009. Comprehensive Mapping of Long-Range Interactions Reveals Folding Principles of the Human Genome. *Science* 326:289–293.
Macé M, Crouau-Roy B. 2008. A highly polymorphic insertion in the Y-chromosome amelogenin gene can be used for evolutionary biology, population genetics and sexing in Cetacea and Artiodactyla. *BMC Genet.* 9:64.
National Center for Biotechnology Information (US). 2017. Gnomon - the NCBI eukaryotic gene prediction tool. Available from: https://www-ncbi-nlm-nih-gov.ezproxy.library.wisc.edu/genome/annotation_euk/gnomon/
Putnam NH, O'Connell BL, Stites JC, Rice BJ, Blanchette M, Calef R, Troll CJ, Fields A, Hartley PD, Sugnet CW, et al. 2016. Chromosome-scale shotgun assembly using an in vitro method for long-range linkage. *Genome Res.* [Internet]. Available from: http://genome.cshlp.org/content/early/2016/02/08/gr.193474.115
Rhie A, McCarthy SA, Fedrigo O, Damas J, Formenti G, Koren S, Uliano-Silva M, Chow W, Fungtammasan A, Kim J, et al. 2021. Towards complete and error-free genome assemblies of all vertebrate species. *Nature* 592:737–746.
Robinson JT, Thorvaldsdóttir H, Winckler W, Guttman M, Lander ES, Getz G, Mesirov JP. 2011. Integrative genomics viewer. *Nat. Biotechnol.* 29:24–26.
Schmieder R, Edwards R. 2011. Quality control and preprocessing of metagenomic datasets. *Bioinformatics* 27:863–864.
Sharma V, Schwede P, Hiller M. 2017. CESAR 2.0 substantially improves speed and accuracy of comparative gene annotation. *Bioinformatics* 33:3985–3987.

Weisenfeld NI, Kumar V, Shah P, Church DM, Jaffe DB. 2017. Direct determination of diploid genome sequences. *Genome Res.* 27:757–767.
Zaharia M, Bolosky WJ, Curtis K, Fox A, Patterson D, Shenker S, Stoica I, Karp RM, Sittler T. 2011. Faster and More Accurate Sequence Alignment with SNAP. *ArXiv11115572 Cs Q-Bio* [Internet]. Available from: http://arxiv.org/abs/1111.5572
